# Meta-Analysis of the Effect of Overexpression of MYB Transcription Factors on the Regulatory Mechanisms of Anthocyanin Biosynthesis

**DOI:** 10.3389/fpls.2021.781343

**Published:** 2021-12-15

**Authors:** Weichao Liu, Tangchun Zheng, Yujie Yang, Ping Li, Like Qiu, Lulu Li, Jia Wang, Tangren Cheng, Qixiang Zhang

**Affiliations:** Beijing Key Laboratory of Ornamental Plants Germplasm Innovation & Molecular Breeding, National Engineering Research Center for Floriculture, Beijing Laboratory of Urban and Rural Ecological Environment, Engineering Research Center of Landscape Environment of Ministry of Education, Key Laboratory of Genetics and Breeding in Forest Trees and Ornamental Plants of Ministry of Education, School of Landscape Architecture, Beijing Forestry University, Beijing, China

**Keywords:** MYB transcription factor, meta-analysis, overexpression, physiological parameter, experimental variable, anthocyanin biosynthesis

## Abstract

MYBs (*v-myb* avian myeloblastosis viral oncogene homologs) are important transcriptional regulators that play critical roles in the regulation of anthocyanin biosynthesis. The overexpression of *MYB* genes has been reported in different plant species. However, the inconsistent strategies to assess transgenic plants have made it difficult to explain the complex mechanisms of regulation of anthocyanin biosynthesis by *MYBs*. We report here a meta-analysis of 608 studies from 206 publications assessing the effects of *MYB* overexpression on anthocyanins and evaluate the experimental variables that have an influence on transgenic plant performance. We found that *MYB* expression enhanced the magnitude of 20 out of 26 examined plant parameters by at least of 21% and reduced the magnitude of 1 indicator by at least 37%. We explored the variety of moderating variables causing these variations. A deeper color induced by *MYBs* caused higher plant attributes as compared to normal color changes. *MYB* genes from dicots stimulated the accumulation of anthocyanins, flavonols and impacted the expressions of *PAL*, *CHS*, *CHI*, *FLS*, *F3′5′H*, *ANS*, *UFGT*, and *ANR* as compared to monocots. Heterologous expression and homologous expression showed a great difference in anthocyanin biosynthesis. Transient gene transformation had a significant effect on the expression of flavonoid biosynthetic genes, and stable transformation had a significant effect on flavonoid accumulation. Stress could result in a significantly increased accumulation of flavonoids, especially anthocyanin, flavonol, and proanthocyanidin. Our study, thus, provides new insights into the function of *MYBs* in the regulatory mechanisms of flavonoid biosynthesis and the use of genetic engineering for improving anthocyanins contents.

## Introduction

Anthocyanins are a flavonoid class of phenylpropanoid compounds and are widely distributed in land plants (Buer et al., 2010; Albert et al., 2014; Xu et al., 2015). Anthocyanin accumulation in vacuoles accounts for the red to blue color range of various tissues, including roots, stems, leaves, flowers and fruits (Ben Zvi et al., 2008; Kim et al., 2010; Fan et al., 2020; Zhong et al., 2020; Xia et al., 2021). They play crucial roles in plant stress resistance by acting as scavengers of excess reactive oxygen species (ROS) (Buer et al., 2010; Naing et al., 2018; Shen et al., 2019; Ma et al., 2021). Therefore, understanding the anthocyanin biosynthesis pathways and the functions of genes that regulate these pathways will contribute to the molecular designing and breeding of plants.

The synthesis of anthocyanins is highly conserved among seed plants, and there is an increasing understanding of the mechanisms that regulate their biosynthesis (Albert et al., 2014; Ma and Constabel, 2019; Li Y. et al., 2020). Anthocyanidin biosynthesis occurs through phenylpropanoid pathway. (Ma and Constabel, 2019). The phenylpropanoid biosynthetic genes at the initial steps include phenylalanine ammonia lyase (PAL), cinnamate 4-hydroxylase (C4H), and 4-coumarate-CoA ligase (4CL) (Fan et al., 2020). Flavonoid biosynthetic genes at early steps include chalcone synthase (CHS), chalcone isomerase (CHI), and flavanone 3-hydroxylase (F3H) (Zhao, 2015). Late biosynthetic genes encode catalytic enzymes, including flavonol synthase (FLS), flavonoid 3′-hydroxylase (F3′H), flavonoid 3′,5′-hydroxylase (F3′5′H), dihydroflavonol 4-reductase (DFR), anthocyanidin synthase (ANS), UDP-flavonoid glucosyl transferase (UFGT), leucoanthocyanidin reductase (LAR) and anthocyanidin reductase (ANR) (Xu et al., 2015). MYB transcription factors (TFs) are the most important genes the anthocyanidin biosynthesis pathway (Yan et al., 2021).

MYB transcription factors possess two distinct regions, a highly conserved MYB DNA-binding domain and a diverse C-terminal modulator region (Ma and Constabel, 2019). Based on the number of repeats (R) in the MYB domain (Buer et al., 2010), MYBs are divided into four classes (1R, R2R3, R1R2R3, and 4R-MYB proteins). The MYB TFs regulate important processes in higher plants, such as secondary metabolism, signal transduction, and biotic and abiotic stresses (Buer et al., 2010; Wang et al., 2019; Ma et al., 2021; Xia et al., 2021). Particularly, they are the vital regulators of anthocyanidin biosynthesis. Many *MYB* genes have been overexpressed in flowers, fruits, vegetables, and crops, frequently to activate anthocyanin accumulation (Dubos et al., 2010; Zhang et al., 2017; Fan et al., 2020; Xu et al., 2020; Ma et al., 2021). Most of the MYB TFs can directly or indirectly activate the promoters of structural genes of flavonoid biosynthesis pathways, thereby affecting the final anthocyanin accumulation (Schaart et al., 2013). In radish (*Raphanus sativus*), RsPAP2 binds to the *RsUFGT* and *RsTT8* promoters and activates the upregulation of anthocyanin-related structural genes, resulting in anthocyanin accumulation (Fan et al., 2020).

Although the anthocyanin biosynthetic pathway are highly conserved in land plants, the overexpression of *MYB* can influences the localization, timing, and optimal intensity of pigment deposition among different plants species. A variety of morphological and biochemical analyses and the expression of flavonoid biosynthetic genes have been used to assess the impact of *MYB* overexpression on the regulatory mechanisms of anthocyanidin biosynthesis. Various types of parameters have been assessed in different studies depending upon the complexity of anthocyanidin biosynthesis pathways. Therefore, an overall evaluation of their size and direction is an important question for investigation. Sample type, overexpression type, expression systems and culture conditions also varied among studies. Thus, an unbiased, comprehensive and comparative analysis of the impacts of a variety of variables from plenty of studies would facilitate our understanding of the influence of *MYB* overexpression on the regulatory mechanisms of anthocyanidin biosynthesis.

Meta-analysis can be used for the statistical analysis of a massive number of datasets obtained from individual researches to determine the effect of the various variables on a number of phenotypic responses (Borenstein et al., 2009; Zhang et al., 2020). Meta-analysis is a powerful and conducive approach to classify and identify biological responses to regulatory genes, including *cation/proton antiporter 1* (*CPA1*) genes, *C-repeat/dehydration responsive element binding proteins* (*CBF/DREB*), and the *NAC* (*NAM/ATAF/CUC*) family (Dong et al., 2017, 2018; Ma et al., 2017; Figueroa et al., 2021). The previous meta-analysis mainly focused on the effect of overexpression of family genes on abiotic stress in plants, but no report has revealed the effect of *MYB* genes overexpression on anthocyanin biosynthesis. We conducted a meta-analysis of *MYB* overexpression to determine their impacts on anthocyanidin biosynthesis in plants. Additionally, thirteen different moderator variables were also investigated to determine which one can influence the extent of the *MYB* effect on anthocyanin accumulation. The main objectives of this study were to answer the following questions: (I) what is the overall effect of *MYB* overexpression in transgenic plants across studies on the transcriptional regulation of anthocyanin biosynthesis? (II) how can specific experimental variables influence the efficacy of *MYB* overexpression? (III) which plant characteristics of anthocyanin biosynthesis were easily affected by *MYB*? This study, thus, summarizes the responses of *MYB*-overexpressing plants to anthocyanin biosynthesis, thereby identifying potential research areas that will allow scholars to better comprehend the impact of MYB transcription factors in the regulation of flavonoid biosynthesis.

## Materials and Methods

### Data Collection

We collected peer-reviewed publications that investigated the impacts of *MYB* overexpression on the regulatory mechanisms of anthocyanidin biosynthesis using the Web of Science and PubMed databases up to 31 December 2020. An extensive literature search was performed for articles dated through 31 December 2020 using our systematic search terms: (*MYB* or MYB transcription factor*) and (overexpression* or ectopic* expression* or transgenic or heterologous) and (anthocyanin* or leucoanthocyanidin*). A total of 496 unique articles were sorted. Upon examination, 290 publications were excluded because they did not conform to the following criteria: the study did not relate to MYB transcription factors (35 articles); *MYB* genes were not overexpressed (112 articles); articles were reviews (13 articles); either treatment or control means were missing (6 articles); or articles did not report the outcome variables of interest (124 articles). Finally, 206 articles spanning 19 years (2001-2020) on *MYB* overexpression in the regulation of the anthocyanin biosynthetic pathway were contained in our meta-analysis. The details are described in Supplementary Table 1 and Supplementary Figure 1.

Multiple treatments from one article were deemed independent studies and represent a unit in the meta-analysis. Means and sample sizes were extracted for both *MYB* overexpression plants and corresponding non-transformed parentals from each of the individual studies. Biological replicates were referred to “sample sizes”. Where articles did not report those sizes, we determined the values conservatively as *n* = 1 if average values were not reported and *n* = 2 if average values were provided. When the sample capacity was given as a scale, the meta-analysis selected the minimum value. When the article included data for a time course, we selected the final time point as separate observation. We used GetData Graph Digitizer^1^ to extract the data in the chart.

### Effect Sizes and Moderators

A meta-analysis was performed on the response of various variables in control and *MYB* transgenic plants to the modulation of anthocyanin accumulation. Each study was included in a meta-analysis, and the combined response of the transgenic plants was defined corresponding to non-transgenic plants to compare a treatment effect size, which was calculated as the natural logarithm of the response ratio (ln *R*) of the transgenic to non-transgenic (control plant means) (Hedges et al., 1999). Following formula was applied:


$$\ln R=lnY_{TC,/}Y_{NC}$$

Here, *Y*_*TC*_ and *Y*_*NC*_ refer to the average values of responses from transformed (TC) plants and corresponding non-transformed (NC) parentals, respectively (Ma et al., 2017). The ln *R*-values are largely applied in meta-analyses of plant behaviors to obtain a standardized, unitless expression of the effects of variables and treatments, revealing biological significance (Borenstein et al., 2009; Ma et al., 2017). Across response ratios, the positive and negative treatment effects are properly balanced by log transformation, which maintains symmetry in the meta-analysis. The ln *R*-values greater than 0 imply an increase in the measured parameter after *MYB* overexpression and the ln *R*-values less than 0 imply that the measured parameter is decreased or unchanged. For ease of interpretation, the mean ln *R* was transformed into the percentage change using the following formula:


$$\mathrm{Percent}\mathrm{change}=\left(e^{\ln R}-1\right)\times 100\%.$$

Overall, we calculated summary effect size for each of the 26 parameters as the weighted average of effect sizes from the initial studies. Apart from the effect size data, thirteen experimental variables or moderator variables was collected from each study. The response to anthocyanidin biosynthesis was modified by these variables. We used moderator variables to test for heterogeneity within the effect sizes. These moderators were of four categories: (I) anthocyanin and color: anthocyanin content change, color, color change, numbers of color types; (II) experimental materials: sample type, taxonomic class (dicot or monocot, or other) and genus of gene donor and recipient, whether the gene donor and recipient were the same genus; (III) character of foreign genes: overexpression type, number of genes transferred; and (IV) experimental conditions: whether experimental conditions were normal culture.

Each moderator variable included more than two levels (categories), and a level of a moderator was required to be documented in no less than three studies, with ≥ 2 published report. Categorical levels of moderators not meeting the stated criteria were classified as “other”. However, the level of “other” was also needed to contain data obtained from no less than three studies, with ≥ 2 published report.

### Meta-Analysis

We used Comprehensive Meta-Analysis (CMA) software (Version 3.0, Biostat, Englewood, NJ) for meta-analyses. Single studies were weighted using non-parametric variance methods:


$$V\ln R=\left(n_{TC}+n_{NC}\right)/\left(n_{TC}*n_{NC}\right)$$

Here, *n*TC and *n*NC refer to the number of samples of the TC and NC samples, respectively, and *V* ln *R* refer to the variance of the natural log of the response ratio *R* (Borenstein et al., 2009). The summary effect was assumed significant if its *p-*value was less than 0.05 and the 95% confidence intervals (CIs) did not overlap 0. We used the *Q* statistic to assess whether the effect value indicated heterogeneity, and to quantify the heterogeneity of the observed effect size. The true heterogeneity of the effect value did not exist when its *I*^2^-value was 0%, while the *I*^2^-value > 50% reflected unacceptable heterogeneity. A common variance among studies across moderator subgroups was supposed when its 95% CI did not overlap 0 and the p value was less than 0.05.

Because statistically significant studies are easy to publish than non-significant studies, and meta-analyses often focus on the published literature, and there can be publication bias in meta-analysis selection bias (Borenstein et al., 2009). Although published articles were accessed from multiple sources to avoid publication bias, it is unclear whether such bias exists. Therefore, we adopted four statistical methods in our study to test potential publication bias, which was quantified using the classic fail-safe N test Begg and Mazumdar (1983), Orwin (1983) rank correlation test, Egger’s regression intercept test (Egger et al., 1997) and Duval and Tweedie’s trim and fill calculation (Duval and Tweedie, 2000).

## Results

### Publication Bias

Within the classic fail-safe N test, seven of the 26 summary effects had a *p-*value ≥ 0.01, indicating that there was concern of bias (Table 1). The Begg and Mazumdar rank correlation test showed that only one summary effect had a *p* < 0.05, indicating the possibility of bias. Egger’s regression intercept test demonstrated that three summary effects may be biased. Egger’s regression intercept test demonstrated that there may be publication bias in three summary effects. Duval and Tweedie’s trim and fill analysis indicated that 22 of these effects may be affected by adjusted value. The adjusted value of flavonoid content, *PAL*, and *ANR* were closer to 0 than the primeval point estimates, while 19 of the 22 summary effects were farther from 0. Therefore, the three summary effects may have a bias effect. Using four quantitative methods, a total of 11 summary effects were evaluated, which may have publication bias. Two summary effects were detected by two methods, and none was detected by three or more methods. This shows that there was no significant bias in 26 summary effects, which was not an important influence on the results of this study.

**TABLE 1:** Publication bias for all the outcome variables included in this study.

Effect size	Summary effect^a^			Classic fail-safe N^b^	Begg and Mazumdar rank correlation^c^		Egger’s regression intercept^d^		Duval and Tweedie’s trim and fill^e^	
					
	N	ln *R*	*P*	*p*	Tau	*p*	intercept	*p*	adjusted	trimmed
Anthocyanin content	469	1.286	0.000	0.000	–0.032	0.305	–1.932	0.008	1.899	90
Cyanidin content	35	2.161	0.000	0.000	0.111	0.349	3.099	0.488	2.833	5
Delphinidin content	16	1.823	0.000	0.000	–0.108	0.558	5508.593	0.197	2.074	2
Flavonoid content	12	0.194	0.417	0.408	0.076	0.732	1.893	0.622	0.012	3
Flavonol content	32	0.444	0.004	0.002	–0.014	0.910	–0.184	0.919	0.726	8
Proanthocyanidin content	78	1.005	0.000	0.000	0.067	0.386	2.760	0.230	1.470	15
Quercetin content	25	0.428	0.007	0.012	–0.130	0.362	–3.943	0.014	0.604	5
Kaempferol content	20	0.570	0.001	0.002	–0.089	0.581	–2.095	0.336	0.793	4
pH	23	–0.031	0.738	0.729	0.211	0.170	–0.047	0.532	–0.031	0
CAT activity	10	0.314	0.224	0.224	0.022	0.929	126.725	0.853	0.350	1
MDA content	15	–0.467	0.027	0.027	–0.095	0.621	–707.784	0.334	–0.467	0
DDPH activity	21	0.493	0.004	0.004	–0.114	0.469	0.153	0.917	1.738	3
*PAL*	86	0.383	0.004	0.000	0.015	0.835	1.391	0.334	–0.133	22
*C4H*	44	0.230	0.257	0.068	0.006	0.952	0.509	0.821	0.230	0
*4CL*	54	0.153	0.420	0.190	0.058	0.536	1.180	0.514	0.380	4
*CHS*	291	1.336	0.000	0.000	–0.016	0.693	–0.185	0.924	1.972	60
*CHI*	243	0.975	0.000	0.000	–0.087	0.043	–3.323	0.010	1.246	55
*F3H*	236	1.238	0.000	0.000	–0.065	0.138	–3.534	0.067	1.858	55
*FLS*	104	0.372	0.006	0.000	–0.045	0.498	–1.137	0.483	0.881	24
*F3′H*	147	1.133	0.000	0.000	–0.013	0.815	0.581	0.741	1.500	21
*F3′5′H*	27	2.284	0.000	0.000	0.028	0.835	2730.129	0.709	2.284	0
*DFR*	330	1.482	0.000	0.000	–0.047	0.202	–3.168	0.166	2.212	63
*ANS*	295	1.633	0.000	0.000	–0.030	0.442	–2.505	0.343	2.331	52
*UFGT*	160	1.021	0.000	0.000	–0.022	0.684	–1.079	0.630	1.523	31
*LAR*	43	0.602	0.016	0.000	0.016	0.884	1.935	0.807	1.319	13
*ANR*	54	0.659	0.000	0.000	–0.005	0.958	–0.201	0.945	0.175	12

*^a^Summary effect: n indicates number of studies from which the summary effect sizes were pooled, ln R represents natural log of the overall summary effect, and p represents probability that the overall summary effect ≠ 0;*

*^b^Classic fail-safe N: p represents P-value of the summary effect sizes, p ≥ 0.01 suggesting publication bias;*

*^c^Begg and Mazumdar Kendall rank correlation: tau indicates rank correlation coefficient (with continuity correction), two-tailed p represents possibility that the sizes of study effect are related to their sampling variances, p < 0.05 indicating publication bias;*

*^d^Egger’s regression intercept: intercept represents intercept of the regression line, two-tailed p represents possibility of significant asymmetry in the study size and study effect size association, p < 0.05 indicating publication bias;*

*^e^Duval and Tweedie trim and fill: adjusted refers to the use of an iterative trim and fill procedure to estimate adjusted summary effect after imputing missing studies; trimmed represents number of studies adjusted using an iterative trim and fill procedure.*

### Heterogeneity Analysis

As shown in Table 2, we found substantial heterogeneity (*P-*value < 0.100) and *I*^2^-value > 50% in 18 out of 26 summary effects values. Therefore, subgroup analysis can be used to search the possible sources of true heterogeneity among studies. In meta-analyses, effect size heterogeneity usually relates to the change in “true” effect sizes. However, heterogeneity and random error make up the “true” observed changes, and a *P-*value > 0.100 or *I*^2^-value < 25% does not essentially mean that real heterogeneity among studies does not exist. Insufficient sample size and large differences in test conditions may also result in a *P-*value > 0.100. Therefore, even if there was no heterogeneity in the statistical significance, we considered selecting a random effect model to perform the subgroup analysis of the summary effect sizes for different variables.

**TABLE 2:** Heterogeneity statistics for the 26 summary effect sizes.

Summary effect size	*Q*-value	*P-*value	*I* ^2^	Change (%)
Anthocyanin content	3107.332	0.000	84.939	262
Cyanidin content	441.518	0.000	92.299	768
Delphinidin content	61.498	0.000	75.609	519
Flavonoid content	4.514	0.952	0.000	21
Flavonol content	36.625	0.224	15.358	56
Proanthocyanidin content	305.151	0.000	74.767	173
Quercetin content	14.613	0.932	0.000	53
Kaempferol content	18.203	0.509	0.000	77
pH	0.639	1.000	0.000	-3
CAT activity	0.799	1.000	0.000	37
MDA content	1.628	1.000	0.000	-37
DPPH activity	6.463	0.998	0.000	64
*PAL*	184.556	0.000	53.943	47
*C4H*	110.312	0.000	61.020	26
*4CL*	141.205	0.000	62.466	17
*CHS*	1975.932	0.000	85.323	280
*CHI*	849.802	0.000	71.523	165
*F3H*	1263.582	0.000	81.402	245
*FLS*	291.320	0.000	64.644	45
*F3′H*	575.052	0.000	74.611	210
*F3′5′H*	317.280	0.000	91.805	882
*DFR*	3086.030	0.000	89.339	340
*ANS*	2964.417	0.000	90.082	412
*UFGT*	702.908	0.000	77.380	178
*LAR*	166.432	0.000	74.765	83
*ANR*	133.382	0.000	60.265	93

*Q-value represents total observed variation among studies; P-value represents probability that Q-value was due to sampling error and not to real variation among true effects; I^2^ is percentage of heterogeneity due to variation among true effects; Changes to summary effect sizes are caused by foreign MYB genes, transformed from ln R to raw percentages. A positive value indicates that MYB overexpression has a promoting effect, and a negative value indicates that MYB overexpression has an inhibitory effect.*

### Overall Summary Effects

**FIGURE 1:**
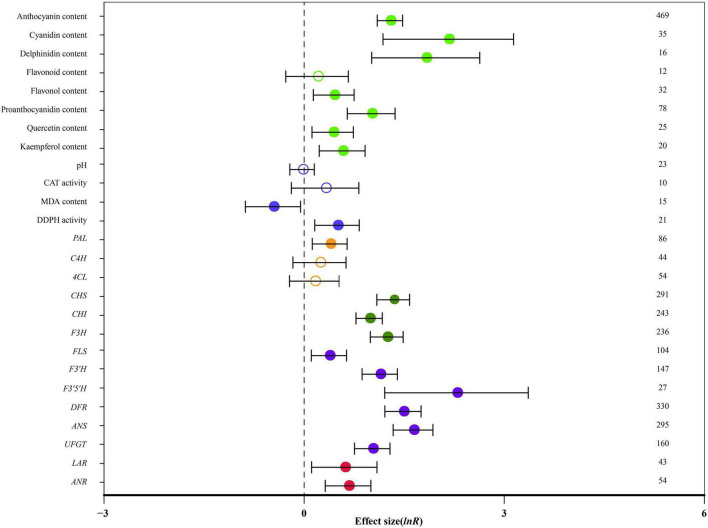
Effect of *MYB* overexpression on the 26 summary effect sizes (natural log of the response ratios of TC/NC plants, ln *R*). Ratios are unit-less. The ratios illustrate the strength of *MYB* overexpression on summary effect sizes compared with the controls. Circles and horizontal bars indicate the summary effects and the 95% CI, respectively. Numbers represent the number of studies contributing to each summary effect. The characteristics of the examined plants are colored and distinguished by different colors. Closed and open circles indicate the effects of significance and insignificance, respectively.

Figure 1 summarizes the average ratio of TC and NC plants of 26 indicators. Seventy-eight species documented in 206 publications were used in the meta-analysis. A total of 59 genera were used as donors, with *Arabidopsis* being the most studied for *MYB* genes, (91 studies), followed by *Malus* (56 studies) and *Solanum* (51 studies). Regarding the type of donors, the most examinations were obtained from dicots (554 studies). The diversity of receptor genera was less and it was observed in only 33 genera; *Nicotiana* was the most studied (225 studies), followed by *Arabidopsis* (123 studies) and *Malus* (52 studies). The highest number of studies (503 studies) was related to stably overexpressing MYB transcription factors and 105 studies discussed transient overexpression. Natural logs (ln *R*) of summary effect sizes are shown in forest plots (Figure 1), where the summary effect size and its CIs are represented in log scale, along with percentage changes calculated from reverse-transformed data.

Overall, *MYB* transformation had a statistically significant impact on 21 out of 26 measured plant parameters (Figure 1: *p* < 0.05, CIs did not cross-zero). The anthocyanin content, cyanidin content and delphinidin content were significantly affected by *MYB* overexpression. Cyanidin content was the most impacted (768%; Table 2). The contents of flavonol, proanthocyanidin, quercetin and kaempferol were increased by more than 53%, while flavonoid content was not affected by overexpressing *MYB*. Transformation did not affect pH or catalase (CAT) activity. In transgenic plants, malondialdehyde (MDA) content was reduced by 37%, whereas the activity of 1,1-diphenyl-2-picryl-hydrazyl (DDPH) was improved by 64% (Table 2). The expression of *PAL* was significantly increased in TC plants by 47%, while the expression of *C4H* and *4CL* did not significantly respond to transformation. The expression of *CHS*, *CHI* and *F3H* was also significantly increased by 165%. *MYB* overexpression had statistically significant impacts on the regulation of late structural genes of anthocyanin biosynthesis, including *FLS*, *F3′H*, *F3′5′H*, *DFR*, *ANS* and *UFGT*. The expression of *F3′5′H* was the most impacted (882%), followed by *ANS* (412%) and *DFR* (340%). The expression of *LAR* and *ANR* were also improved by *MYB* transformation (Table 2).

### Subgroup Analysis

**FIGURE 2:**
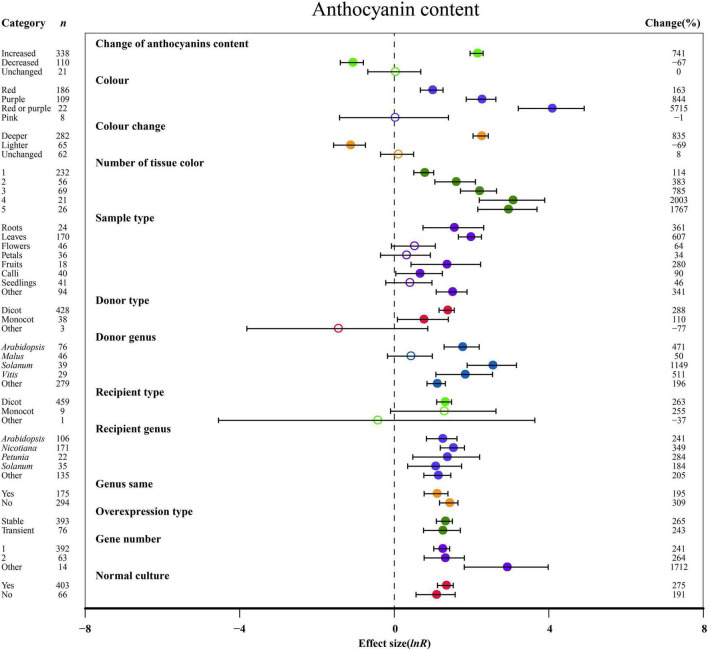
Summary effects (ln *R*) of the influence of *MYB* overexpression on anthocyanin content in plants. Ratios are unit-less. The ratios illustrate the strength of *MYB* overexpression on the summary effects of anthocyanin content compared with the controls. Circles and horizontal bars indicate the summary effects and the 95% CI of thirteen moderator variables, respectively. *n* represents the number of studies. Subgroup list levels (subgroups or categories) of each moderator. The percent change (to the right of plots) refers to the raw percentage increase or decrease in anthocyanin content induced by overexpression of *MYB*. The moderator variables of the examined plants are colored and distinguished by different colors. Closed and open circles indicate the effects of significance and insignificance, respectively.

**FIGURE 3:**
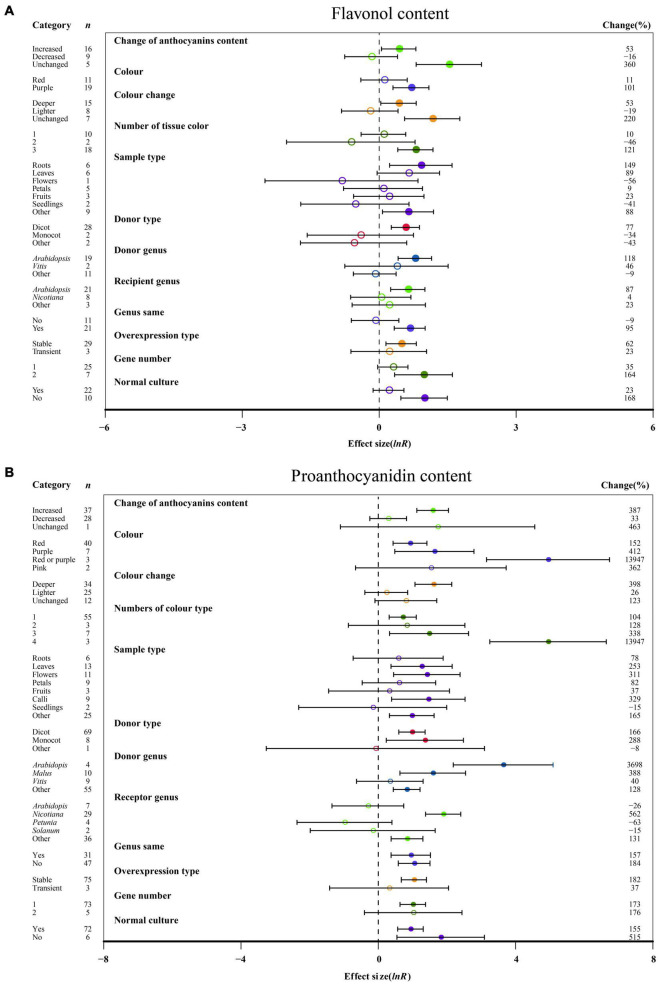
Summary effects (ln *R*) of the influence of *MYB* overexpression on the contents of flavonol **(A)** and proanthocyanidin **(B)** in plants. Ratios are unit-less. The ratios illustrate the strength of *MYB* overexpression on the summary effects of the flavonol and proanthocyanidin contents compared with the controls. Circles and horizontal bars indicate the summary effects and the 95% CI of twelve **(A)** owing to insufficient studies, “recipient type” not analyzed) and twelve **(B)** owing to insufficient studies, some moderators not analyzed) moderator variables, respectively. *n* represents the number of studies. Subgroup list levels (subgroups or categories) of each moderator. The percent change (to the right of plots) refers to the raw percentage increase or decrease in the content of flavonol and proanthocyanidin induced by overexpression of *MYB*. The moderator variables of the examined plants are colored and distinguished by different colors. Closed and open circles indicate the effects of significance and insignificance, respectively.

**FIGURE 4:**
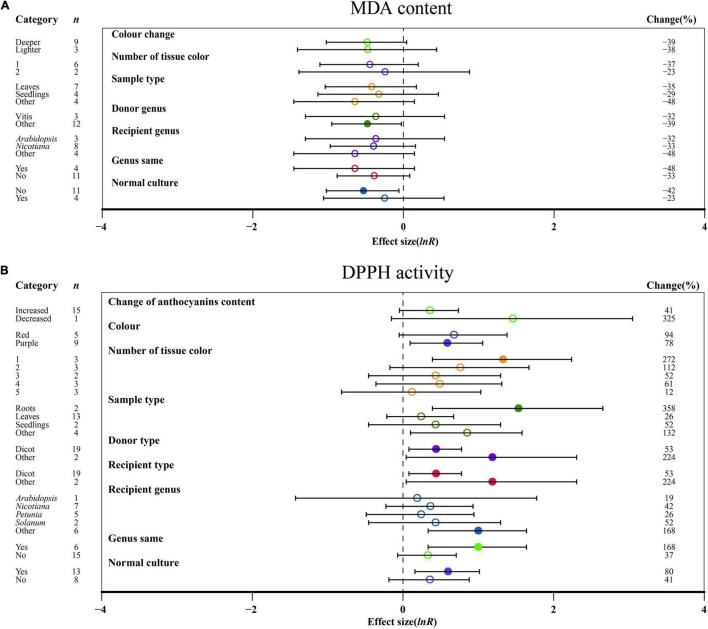
Summary effects (ln *R*) of *MYB* overexpression on MDA content **(A)** and DDPH activity **(B)** in plants. Ratios are unit-less. The ratios illustrate the strength of *MYB* overexpression on the summary effects of MDA content and DDPH activity compared with the controls. Circles and horizontal bars indicate the summary effects and the 95% CI of seven **(A)** owing to insufficient studies, some moderators not analyzed) and nine **(B)** owing to insufficient studies, some moderators not analyzed) moderator variables, respectively. *n* represents the number of studies. Subgroup list levels (subgroups or categories) of each moderator. The percent change (to the right of plots) refers to the raw percentage increase or decrease in MDA content and DDPH activity induced by overexpression of *MYB*. The moderator variables of the examined plants are colored and distinguished by different colors. Closed and open circles indicate the effects of significance and insignificance, respectively.

**FIGURE 5:**
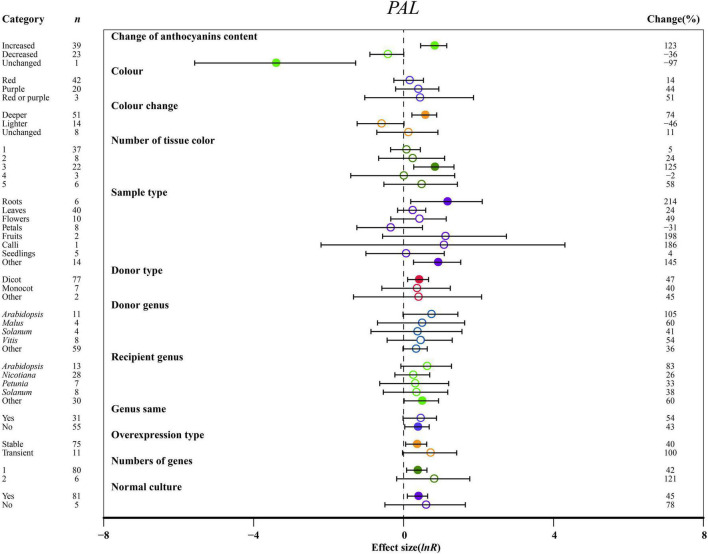
Summary effects (ln *R*) of the influence of *MYB* overexpression on *PAL* expression in plants. Ratios are unit-less. The ratios illustrate the strength of *MYB* overexpression on the summary effects of *PAL* expression compared with the controls. Circles and horizontal bars indicate the summary effects and the 95% CI of twelve (owing to insufficient studies, “recipient type” not analyzed) moderator variables, respectively. *n* represents the number of studies. Subgroup list levels (subgroups or categories) of each moderator. The percent change (to the right of plots) refers to the raw percentage increase or decrease in *PAL* expression induced by overexpression of *MYB*. The moderator variables of the examined plants are colored and distinguished by different colors. Closed and open circles indicate the effects of significance and insignificance, respectively.

**FIGURE 6:**
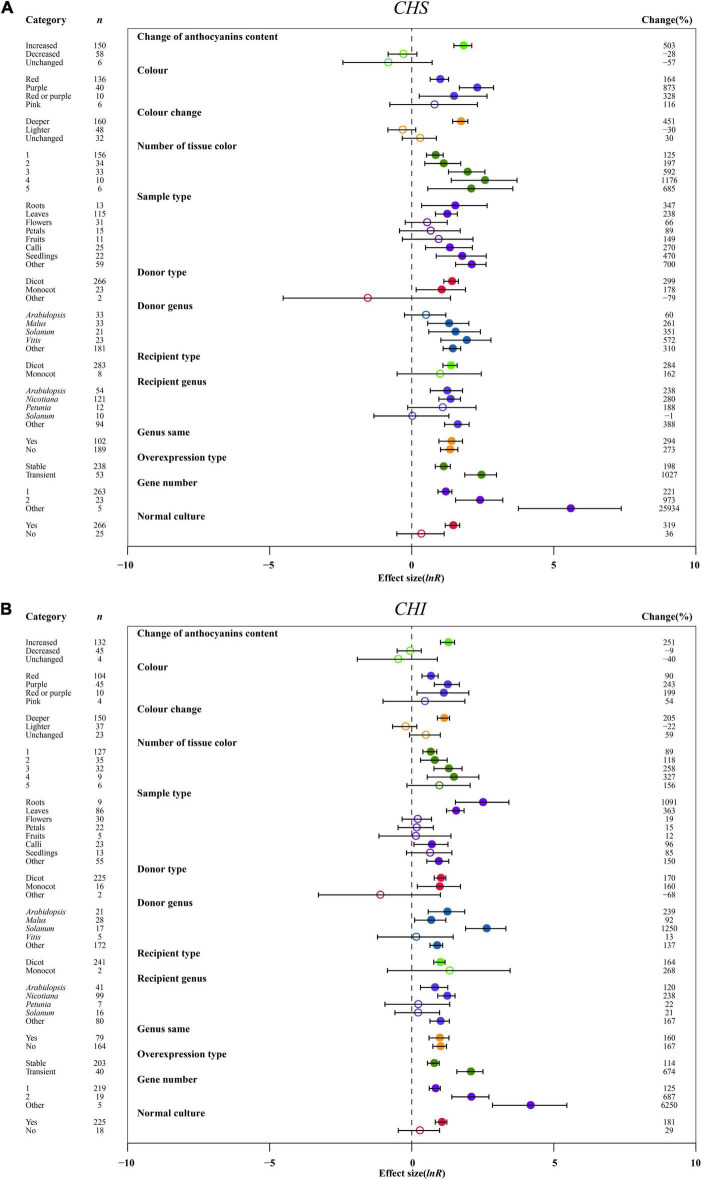
Summary effects (ln *R*) of the influence of *MYB* overexpression on the expression of *CHS* **(A)** and *CHI* **(B)** in plants. Ratios are unit-less. The ratios illustrate the strength of *MYB* overexpression on the summary effects of the expression of *CHS* and *4HI* compared with the controls. Circles and horizontal bars indicate the summary effects and the 95% CI of thirteen moderator variables, respectively. *n* represents the number of studies. Subgroup list levels (subgroups or categories) of each moderator. The percent change (to the right of plots) refers to the raw percentage increase or decrease in the expression of *C4H* and *4CL* induced by overexpression of *MYB*. The moderator variables of the examined plants are colored and distinguished by different colors. Closed and open circles indicate the effects of significance and insignificance, respectively.

**FIGURE 7:**
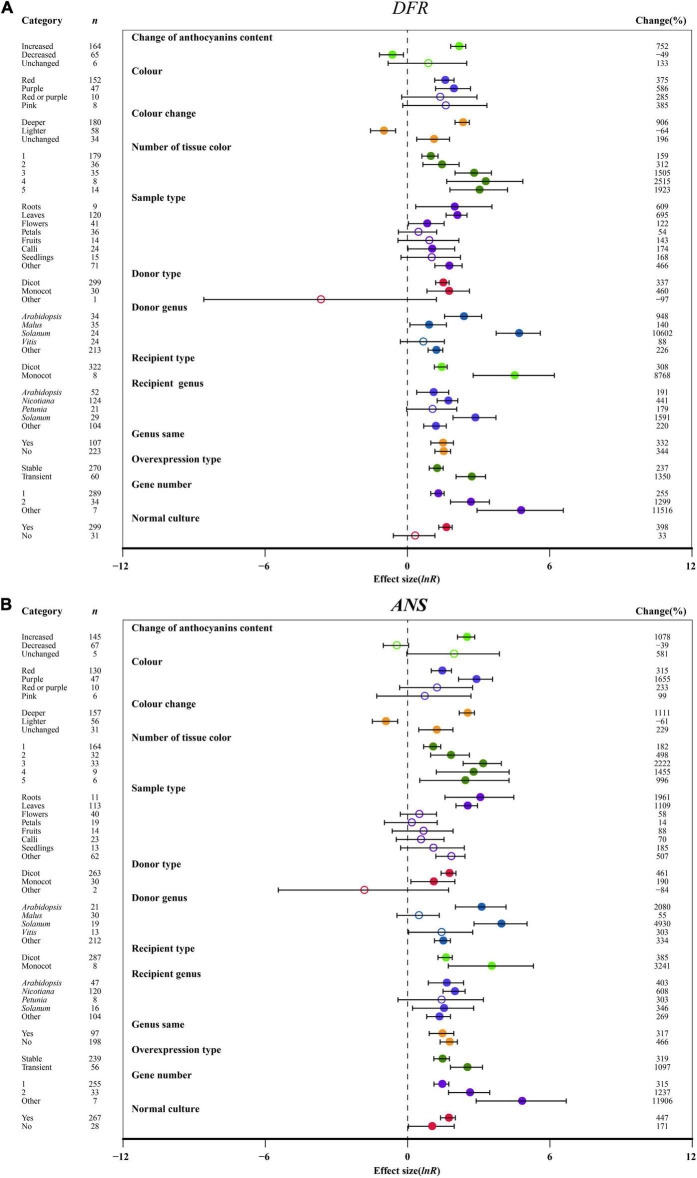
Summary effects (ln *R*) of the influence of *MYB* overexpression on the expression of *DFR* **(A)** and *ANS* **(B)** in plants. Ratios are unit-less. The ratios illustrate the strength of *MYB* overexpression on the summary effects of the expression of *DFR* and *ANS* compared with the controls. Circles and horizontal bars indicate the summary effects and the 95% CI of thirteen moderator variables, respectively. *n* represents the number of studies. Subgroup list levels (subgroups or categories) of each moderator. The percent change (to the right of plots) refers to the raw percentage increase or decrease in the expression of *DFR* and *ANS* induced by overexpression of *MYB*. The moderator variables of the examined plants are colored and distinguished by different colors. Closed and open circles indicate the effects of significance and insignificance, respectively.

**FIGURE 8:**
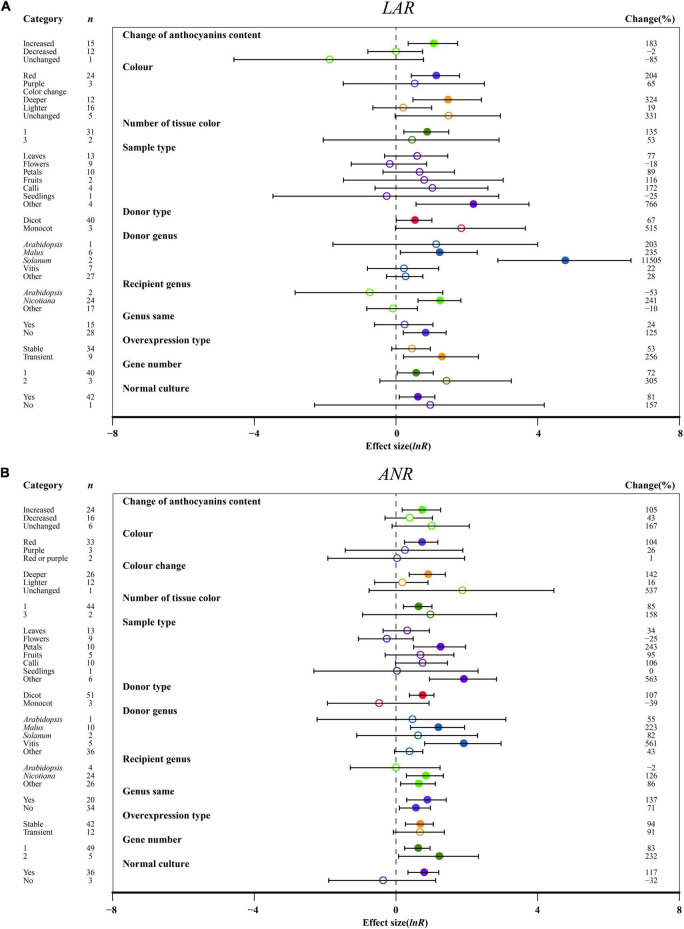
Summary effects (ln *R*) of the influence of *MYB* overexpression on the expression of *LAR* **(A)** and *ANR* **(B)** in plants. Ratios are unit-less. The ratios illustrate the strength of *MYB* overexpression on the summary effects of the expression of *LAR* and *ANR* compared with the controls. Circles and horizontal bars indicate the summary effects and the 95% CI of eleven **(A)** owing to insufficient studies, some moderators not analyzed) and twelve **(B)** owing to insufficient studies, “recipient type” not analyzed) moderator variables, respectively. *n* represents the number of studies. Subgroup list levels (subgroups or categories) of each moderator. The percent change (to the right of plots) refers to the raw percentage increase or decrease in the expression of *LAR* and *ANR* induced by overexpression of *MYB*. The moderator variables of the examined plants are colored and distinguished by different colors. Closed and open circles indicate the effects of significance and insignificance, respectively.

By performing subgroup analysis of the 26 summary effects, we determined the categories or levels variables with significant effects on true heterogeneity. Moderating variables such as experimental conditions, experimental materials and characteristics of foreign genes were registered and used for subgroup analysis. Figures 2–8 and Supplementary Figures 2–8 show the effect of the moderator levels (categories) on the summary effects. We found substantial heterogeneity (*P-*value < 0.100) and *I*^2^-value > 50% in 18 out of 26 summary effect values (Table 2). In meta-analyses, the heterogeneity of the effect values usually refers to the change in “true” effect sizes and the change in “true” effect sizes usually represents the heterogeneity of the effect sizes.

However, heterogeneity and random error make up the “true” observed changes, and a *P-*value > 0.100 or *I*^2^-value < 25% does not essentially mean the absence of real heterogeneity among studies (Borenstein et al., 2009). Insufficient sample size and large differences in test conditions may also result in a *P-*value > 0.100. Therefore, even if there was no heterogeneity in the statistical significance, we considered selecting a random effect model to conduct the subgroup analysis of the summary effect sizes for different variables.

### Subgroup Analysis of Flavonoid Contents

The overexpression of *MYB* in transgenic plants strongly promoted the contents of anthocyanin, cyanidin, delphinidin, flavonol, proanthocyanidin, quercetin and kaempferol (Figures 2, 3 and Supplementary Figures 2, 3). Compared with the decreased or unchanged anthocyanin content, the transgenic plants showed significant increase in the contents of anthocyanin, cyanidin and proanthocyanidin to 741, 18,468, and 387%, respectively (Figure 2 and Supplementary Figure 2A). When the anthocyanin content was unchanged, the transgenes had the greatest impact on the flavonol content (Figure 3A). However, the change in anthocyanin content did not show significant penalties in the contents of quercetin and kaempferol (Supplementary Figure 3). When the color of transgenic plant tissue was red or purple, the effects of *MYB* transformation on the contents of anthocyanin, cyanidin, and proanthocyanidin were more significant than those of other colors (Figures 2, 3B and Supplementary Figure 2A). In the red tissues, the contents of delphinidin and kaempferol were significantly improved by 115 and 111%, respectively (Supplementary Figures 1B, 3B). In the purple tissues, the *MYB* transformation had positive effects on the contents of flavonol and quercetin (Figure 3A and Supplementary Figure 3A). The cyanidin content was the most impacted, reaching 32.4×, followed by the anthocyanin content (8.4×) and delphinidin content (5.6×) (Figures 2, 3 and Supplementary Figures 2, 3). However, during a lighter color change, *MYB* transformation negatively impacted the anthocyanin content and cyanidin content, decreasing up to 69% and 98%, respectively (Figure 2 and Supplementary Figure 2A). However, when the color was unchanged, NC plants improved delphinidin content and flavonol content by 29.9× and 2.2×, respectively (Figure 3A and Supplementary Figure 2B).

With an increasing number of tissue colors in transgenic plants, the accumulation of anthocyanin content and cyanidin content increased significantly (Figure 2 and Supplementary Figure 2A). If the tissue number was 4, MYB transformation had the maximum effect on cyanidin and proanthocyanidin contents, which was more than sixteen times and thirty-two times that of the other number groups (Figure 3B and Supplementary Figure 2A). Significantly increased anthocyanin, cyanidin, and delphinidin contents were observed in the leaf tissues of transgenic plants as compared to other tissues (Figure 2 and Supplementary Figure 2). However, increased contents of flavonol, proanthocyanidin, quercetin and kaempferol contents were observed in calluses, roots, seedlings and flowers, respectively (Figure 3 and Supplementary Figure 3). Anthocyanin content and flavonol content were affected by the taxonomy of donor plants *MYB* genes from dicots showed significantly higher responses than those from other donor plants (Figures 2, 3A). Contrarily, Monocot donors showed 1.7 times higher contents of proanthocyanidin as compared to dicots (Figures 2, 3B). For receivers, dicots accumulated more anthocyanin content than monocot plants (Figure 2). Regarding the genera of donors, TC plants showed the maximum improvement in the contents of cyanidin, flavonol, proanthocyanidin and kaempferol for overexpressing *MYB* genes from *Arabidopsis*, whereas the most anthocyanin and delphinidin content responses were observed for *Solanum* (Figures 2, 3 and Supplementary Figures 2A, 3B). *Nicotiana* seems to be an appropriate *MYB* recipient genus, imparting at least 562% increase in the contents of anthocyanin, cyanidin, delphinidin and proanthocyanidin (Figures 2, 3A and Supplementary Figure 2). However, the flavonol, quercetin and kaempferol contents of recipient *Arabidopsis* were higher than those of other genera (Figure 3A and Supplementary Figure 3). When *MYB* genes were expressed homologously, TC plants showed a better effect on the content of cyanidin, flavonol and quercetin than heterologous expression (Figure 3A and Supplementary Figures 2A, 3A). In contrast, heterologous expression had a significant influence on the responses of the contents of anthocyanin, delphinidin, proanthocyanidin and kaempferol (Figures 2, 3B and Supplementary Figures 2B, 3B). Stable expression of *MYB* genes in plants better affected the contents of anthocyanin, cyanidin, flavonol, proanthocyanidin and quercetin than transient overexpression (Figures 2, 3 and Supplementary Figures 2A, 3A). Transfer of transient cells demonstrated a remarkable TC-induced improvement in the content of delphinidin and kaempferol, which was more than 21-fold and 3-fold than that of stable overexpression (Supplementary Figures 2B, 3B). Transfer of single genes had a greater impact on the content of delphinidin, proanthocyanidin, quercetin and kaempferol than multiple gene transfers (Figure 3B and Supplementary Figures 2B, 3). When two genes were transferred, the accumulation of cyanidin content and flavonol content were markedly increased, wherein the cyanidin content was eight times higher than that of single gene transformation (Figure 3A and Supplementary Figure 2A). More than three genes caused more anthocyanin content, which was more than 5.3 times that of single or two gene transfers (Figure 2). For environmental conditions, compared with normal culture, the positive effects of overexpressing *MYB* on flavonol and proanthocyanidin content were increased when grown in abnormal environments, whereas higher responses of anthocyanin and cyanidin contents were shown in normal environments (Figures 2, 3 and Supplementary Figure 2A).

### Effect of Moderators on Antioxidant Responses

No significant impact of DDPH activity on the change in anthocyanin content was demonstrated in TC plants (Figure 4B). For color, a purple color had a significant influence on DDPH activity after *MYB* transformation than that of red, while MDA content was not significantly altered by the color change (Figure 4). Regarding the number of colored tissues, one tissue provided the highest DPPH activity, which was improved by 272% (Figure 4B). The outcomes of transformation on DDPH activity were most evident in relation to the sampled tissues, with the TC-induced impact averaging 358 and 132% for roots and other tissues, respectively (Figure 4B). As donor plants, other donors showed more DDPH activity after transformation than dicots, with other genera showing the lowest MDA content when acting as donors (Figure 4). Similar to donor plants, DDPH activity was also induced when receiver plants were other receivers (Figure 4B). For the receptor genus, other genera showed a significant increase in DDPH activity relative to *Arabidopsis*, *Nicotiana*, *Petunia*, and *Solanum* (Figure 4B). Homologous expression resulted in higher DDPH as compared to heterologous systems (Figure 4B). When plants were grown in normal culture, the effect of *MYB* transformation on increasing the activity of DDPH was greater, whereas higher inhibition of MDA content was observed in an abnormal environment (Figure 4).

### Effect of Moderators on Phenylalanine Ammonia Lyase, Cinnamate 4-Hydroxylase, and 4-Coumarate-CoA Ligase

Overexpressing *MYBs* significantly upregulated the expression of *PAL* and *C4H*, resulting in the increased anthocyanin content of transgenic plants by at least 2.1-fold as compared to NC plants (Figure 5 and Supplementary Figure 4A). When the anthocyanin content was decreased, *4CL* was downregulated by 63% in *MYB* transformation plants (Supplementary Figure 4B). The highest impact of *MYB* transformation on the downregulation of *C4H* was observed in red tissues of transgenic plants (Supplementary Figure 4A). Regarding the color change, *PAL* and *4CL* were significantly upregulated by a deeper color of TC plants. However, the transcripts of *C4H* and *4CL* decreased gradually due to reduction in color intensity by overexpression of *MYB* (Figure 5 and Supplementary Figure 4). The *PAL* gene was notably expressed in three tissue colors, whereas *C4H* and *4CL* were not significantly changed by the number of color types (Figure 5 and Supplementary Figure 4).

The effects of transformation on *PAL* and *C4H* were the most significant in roots (Figure 5 and Supplementary Figure 4A). In other tissues, *PAL*, *C4H*, and *4CL* were upregulated at least 2.4-fold relative to NC plants (Figure 5 and Supplementary Figure 4). *C4H* expression was rapidly decreased in petals (Supplementary Figure 4A). For donors, dicot plants were much more responsive to the expression of *PAL* than monocotyledonous plants, but donor plants did not significantly differ in *C4H* and *4CL* expression (Figure 5 and Supplementary Figure 4). Other genera showed the highest increase in the transcription of *C4H* in donor plants (Supplementary Figure 4A). Although the expression of *PAL* and *C4H* was significantly induced in transformed *Arabidopsis* and other genera, *C4H* was inhibited when genes came from *Petunia*, or when donor and recipient plants were of the same genera, and *MYB* transformation significantly promoted the expression of *C4H* and *4CL* (Figure 5 and Supplementary Figure 4). However, overexpressing *MYB* in heterologous systems positively regulated *PAL* expression (Figure 5). There was no notable difference between transient transformation and stable transformation in the expression levels of *PAL*, *C4H* and *4CL* (Figure 5 and Supplementary Figure 4). For *PAL* expression, single gene transformation caused a considerable impact on TC plants, and normal culture was a more influential environmental condition than stress (Figure 5).

### Effect of Moderators on Early-Stage Genes of Anthocyanin Biosynthesis

The effect of transformation on improving the expression levels of early-stage genes of anthocyanin biosynthesis was consistent across anthocyanin content changes. The expression of *CHS*, *CHI*, and *F3H* was the highest, causing the increased anthocyanin content of TC plants (Figure 6 and Supplementary Figure 5). For the color change of transgenic plant organs, we also noted a similar result from the early-stage genes, with the greatest effect in purple color, followed by red or purple and red (Figure 6 and Supplementary Figure 5). Similarly, in the anthocyanin content change, the dark color of overexpression plants significantly enhanced the expression of *CHS*, *CHI* and *F3H*, by 5. 5-, 3. 0-, and 4.2-fold, respectively (Figure 6 and Supplementary Figure 5). The expression levels of *CHS*, *CHI* and *F3H* were found to increase with increasing number of organ colors in TC plants, with the highest expression of early-stage genes in four tissue colors (Figure 6 and Supplementary Figure 5). In roots of TC plants, the expression of *CHI* and *F3H* was markedly upregulated by 11.9- and 10.2-fold, respectively, while *CHS* expression was the highest in other tissues (Figure 6 and Supplementary Figure 5). The effect of transformation on the influence of *CHS* and *CHI* expression was consistent across donors and receiver type, with their transcription being higher in dicots (Figure 6). In contrast, *F3H* expression was markedly induced when donors and receivers were monocot plants (Supplementary Figure 5). With *MYB* genes from *Vitis*, the donor genus had similar positive impacts on the expression of *CHI* and *F3H*, upregulated by 13.5- and 80.0-fold, respectively, as compared to genes from solanum (Figure 6B and Supplementary Figure 5). However, *Vitis* seems to be a useful *MYB* donor, causing at least 6.7-fold increase in *CHS* expression (Figure 6A). Although there was no contrasting effect on the expression of *CHS*, *CHI* and *F3H* under recipient *Petunia* and *Solanum*, some differences can be distinguished in *Arabidopsis*, *Nicotiana* and other genera (Figure 6 and Supplementary Figure 5). Although the expression systems of plants considerably enhanced the expression of *CHS*, *CHI* and *F3H*, heterologous or homologous expression demonstrated little difference among genes (Figure 6 and Supplementary Figure 5). Regarding overexpression type, the expression levels of all three genes were improved more than 3.6-fold when *MYB* genes were transiently expressed compared to stable transformation (Figure 6 and Supplementary Figure 5). Consistent with the large accumulation of anthocyanins in transgenic plants, *CHS*, *CHI* and *F3H* expression was significantly activated with increasing gene numbers (Figure 6 and Supplementary Figure 5). Compared to various stresses, normal culture can significantly strengthen the transcriptional levels of *CHS*, *CHI* and *F3H* in transgenic plants (Figure 6 and Supplementary Figure 5).

### Effect of Moderators on Late-Stage Genes of Anthocyanin Biosynthesis

Regarding the change in the anthocyanin contents of the transgenic plants, the expression of *F3′H*, *F3′5′H*, *DFR*, *ANS* and *UFGT* in anthocyanin accumulation was significantly modulated by 4. 7-, 8. 2-, 8. 5-, 11.8- and 3.6-fold, respectively, relative to wild-type plants. But *FLS* expression was not significantly controlled by anthocyanin content change (Figure 7 and Supplementary Figures 6–8). A purple color provided the highest *F3′H*, *F3′5′H*, *DFR*, *ANS* and *UFGT* expression after overexpressing MYB, while the expression of *FLS* was not affected (Figure 7 and Supplementary Figures 6–8). With the exception of *FLS* expression, a deeper color of overexpression plants resulted in a significant enhancement in the expression of the late-stage genes (*F3′H*, *F3′5′H*, *DFR*, *ANS*, *UFGT*), which was upregulated by at least 4.8-fold compared to the NC plants (Figure 7 and Supplementary Figures 6–8). Correspondingly, the transcriptional levels of *F3′5′H*, *DFR* and *ANS* were significantly decreased by the light color of TC plants (Figure 7 and Supplementary Figure 7). We found that the expression of anthocyanin-specific biosynthesis genes (*F3′H*, *F3′5′H*, *DFR*, *ANS*, and *UFGT*) was associated with the number of visual tissues of overexpression plants, and multiple tissues resulted in the transcriptional level of *F3′5′H*, *ANS*, and *UFGT* having a greater effect than one (Figure 7 and Supplementary Figures 6B, 7, 8). *F3′H*, *F3′5′H*, *DFR*, and *UFGT* showed the highest expression levels across roots in TC plants, while *FLS* and *ANS* exhibited higher expression in petal tissues and leaves, respectively (Figure 7 and Supplementary Figures 6–8).

Regarding donor plants, *FLS*, *F3′5′H*, *ANS* and *UFGT* were significantly upregulated by *MYB* genes from monocot plants compared to dicots, but *F3′H* and *DFR* showed the opposite expression pattern (Figure 7 and Supplementary Figures 6–8). Overexpressing *MYB* genes from *Solanum* caused the upregulation of *F3′H*, *DFR*, *ANS* and *UFGT* expression by 39. 1-, 107. 0-, 50. 3-, and 16.2-fold, respectively (Figure 7 and Supplementary Figures 6B, 8). The transcription of *F3′H*, *DFR* and *ANS* was induced in monocot receiver plants (Figure 7 and Supplementary Figure 6B). In the transgenic *MYB*-overexpressing *Solanum*, the expression of *F3′5′H*, *DFR* and *UFGT* relative to NC plants was the most upregulated by 39. 6-, 16. 9-, and 10.1-fold, respectively (Figure 7A and Supplementary Figures 7, 8). The effects of *MYB* overexpression on *FLS*, *F3′H*, *F3′5′H*, *DFR*, *ANS* and *UFGT* expression depends on the expression system (Figure 7 and Supplementary Figures 6–8). *FLS*, *F3′H* and *UFGT* were significantly upregulated in homologous systems compared with heterologous systems, but the opposite expression pattern was shown by *F3′5′H*, *DFR* and *ANS* (Figure 7 and Supplementary Figures 6–8). Additionally, transient transfection of plants seemed to directly correlate with anthocyanin biosynthetic structural genes (*FLS*, *F3′H*, *F3′5′H*, *DFR*, *ANS*, *UFGT*), wherein *F3′5′H* and *DFR* expression was more than 4.3- and 2.9-fold higher than that of stably transformed plants (Figure 7 and Supplementary Figures 6–8). Consistent with the large accumulation of anthocyanins in transgenic plants, multiple gene transfers induced more *F3′H*, *F3′5′H*, *DFR*, *ANS*, and *UFGT* expression (Figure 7 and Supplementary Figures 6B, 7, 8). Under culture conditions, normal culture significantly increased the transcriptional levels of *FLS*, *F3′H*, *F3′5′H*, *DFR*, *ANS*, and *UFGT* in transgenic plants (Figure 7 and Supplementary Figures 6–8).

### Effect of Moderators on Leucoanthocyanidin Reductase and Anthocyanidin Reductase

The relative expression levels of *LAR* and *ANR* were significantly upregulated, causing increased anthocyanin content in transgenic plants as compared to other anthocyanin content changes (Figure 8). Deep colors, such as red, induced higher expression of *LAR* and *ANR* than light colors (Figure 8). One organ color in transgenic plants provided higher expression of *LAR* and *ANR* than multiple tissues, which is different from anthocyanins (Figure 8). Regarding the sampled organs, *ANR* and *LAR* showed the highest expression levels across other tissues in transgenic plants overexpressing *MYB* (Figure 8). For donor plants, *LAR* and *ANR* were notably upregulated in *MYB* genes from dicot plants compared with monocot plants (Figure 8). Interestingly, overexpressing *MYB* genes from *Solanum* showed the highest impacts on *LAR*, and significant impacts of *ANR* expression were observed for *Vitis* (Figure 8). Recipient plants and the type of expression systems were also important. Higher *LAR* and *ANR* expression were observed in the transgenic *MYB*-overexpressing *Nicotiana*, but a large difference in those genes was demonstrated by heterologous or homologous expression (Figure 8). The effect of transformation on the expression of *LAR* and *ANR* was not consistent across overexpression type and number of gene transfers. The *LAR* expression was high in transient transfection and single gene transformation and the *ANR* expression was high in stable overexpression and two genes of TC plants (Figure 8). Growth conditions were also found to alter the expression levels of *LAR* and *ANR*, which were significantly upregulated in transgenic plants grown in a normal environment (Figure 8).

## Discussion

Extensive studies on MYB transcription factors draws attention to their pivotal roles in the regulation of anthocyanins, flavor, and texture. Therefore, we conducted an integrative meta-analysis of a large number of studies investigating the responses of *MYB* overexpression in TC plants in anthocyanin biosynthesis. We employed this method to quantitatively integrate important plant characteristics, including flavonoids, antioxidant responses, and flavonoid biosynthetic genes. In addition to confirming the impact of *MYB* overexpression on anthocyanin biosynthesis, the meta-analysis evaluated the most conducive plant groups and experimental circumstances for the analysis of MYB functions.

The flavonoid pathway produces anthocyanins, proanthocyanidin and other flavonoids. Numerous reports indicate that *MYB* overexpression may also influence the synthesis of other flavonoids while promoting the accumulation of anthocyanins (Shen et al., 2019; Zhong et al., 2020). Consistent with this, our meta-analysis suggests that *MYB* overexpression in transgenic plants promotes flavonoid biosynthetic genes, resulting in the production of more flavonoids, including anthocyanin, cyanidin, delphinidin, flavonol, proanthocyanidin, quercetin and kaempferol. Moreover, the transcriptional regulation of flavonoid pathway by MYBs has become important model for understanding the control of metabolic pathways in plants (Zhou et al., 2020). In addition, TC plants indicated a more than 64% improvement in DPPH activity and a more than 37% decrease in MDA content. These results demonstrate that MYB overexpression may play a key role in plant stress response with the accumulation of anthocyanins (Shen et al., 2019; Zhou et al., 2020).

TC-induced effects on plant attributes occurred with changes in the anthocyanins content and color of transgenic plants. A deep color induced higher plant attributes than light color changes, and similar results were achieved in the accumulation anthocyanin content. Therefore, we can measure the transgenic effect by the color change of *MYB* overexpression in transgenic tissues and organs, thus, proposing the use of *MYBs* as a visual selectable marker for developing plant intragenic vector systems. Furthermore, this method not only provides visualization of the choice of transformed organs but also promotes potential consumption value by anthocyanin accumulation in fruit, vegetables, and berries (Kim et al., 2010; Jin et al., 2012; Allan and Espley, 2018). Kim et al. (2010) suggested that the *IbMYB1a* gene was a selectable marker for sweet potato transformation, because *IbMYB1a* overexpression induced massive anthocyanins. Therefore, the use of *MYBs* can provide a potential alternative to bacterial antibiotic or herbicide resistance genes, which positively affects public acceptance of genetic modification (Jin et al., 2012). This should be a fertile area for future research.

Anthocyanins are flavonoids that are responsible for the diverse colors of roots, leaves, flowers and fruits, including pink, red, purple and blue (Allan and Espley, 2018; LaFountain and Yuan, 2021). This wide range of pigmentation requires the regulation of anthocyanin biosynthesis pathway to enable appropriate localization, timing, and optimal intensity of anthocyanin pigmentation in various organs (LaFountain and Yuan, 2021). The R2R3-MYB TFs are known as the determinants of variation in pigment deposition and color intensity (Xu et al., 2020). *MYB* overexpression promotes the expression of anthocyanin synthesis genes in different tissues and organs of different plants, causing anthocyanins accumulation for various colors. Thus, anthocyanins are usually regulated by MYB transcription factors that have tissue-specific expression profiles (Park et al., 2015; Xu et al., 2020). Consistent with this, the meta-analytic indicates that *MYB* transformation produces anthocyanin accumulation in different tissues of transgenic plants, resulting in a color change. TC plants expressing *DcMYB113* driven by the *CaMV 35S* promoter form purple roots and petioles in carrots, while plants carrying *DcMYB113* driven by its own promoter form purple roots and non-purple petioles, suggesting that promoter determines the root-specific expression of *DcMYB113* (Xu et al., 2020). The anthocyanin accumulation in different organs was increased when *MYB* expression was driven by the *CaMV 35S* promoter than with other promoters (Fan et al., 2020; Xu et al., 2020). Unfortunately, the low study numbers on the use of promoter in MYB transgenic plants show their fewer efficacies for subgroup analysis. Therefore, it seems reasonable to use promoters in overexpression of *MYB* genes to make plant organs produce tissue-specific effects suitable for research purposes, which is a worthy hypothesis for future research.

The extensive characterization of regulatory mechanisms of flavonoids suggests the conserved role of MYB TFs in the regulatory pathways (Zhang et al., 2018; Li Y. et al., 2020; LaFountain and Yuan, 2021). However, it has been indicated that the anabolism of flavonoids, especially anthocyanin biosynthesis in dicots, is possibly different from that in monocots (Li Y. et al., 2020). For example, the regulatory mechanisms of anthocyanin were highly similar in *Freesia hybrida*, *Arabidopsis* and tobacco, but the differences exist in the transactivation capacity by MYB regulators (Li Y. et al., 2020). Moreover, the meta-analysis shows that *MYB* genes from dicot plants showed the highest improvement of anthocyanins, flavonol, and some anthocyanin synthesis genes compared with monocots. It may be that most of the studies reported dicotyledonous plants, with fewer on monocots. Therefore, it is also necessary to study monocots, which can help to understand regulatory differences between MYB regulatory factors of different angiosperms.

The identity of the donor and receptor plants influenced the efficacy of *MYB* transformation on each effect size. *Arabidopsis* is the most studied model system (Ma et al., 2017; Figueroa et al., 2021), providing independent and original research on anthocyanidin biosynthesis (Fan et al., 2020; Zhou et al., 2020). However, the meta-analysis demonstrates that 92 studies came from *Arabidopsis*, which was the largest donor genus but not the best. Compared to other donor plants, overexpressing *MYB* genes from *Solanum* showed the highest improvement in the contents of anthocyanins, delphinidins, and the regulatory genes, such as *CHI*, *F3H*, *F3′H*, *DFR*, *ANS*, and *LAR*. It was speculated that *MYBs* from *Solanum* play a key role in anthocyanin biosynthesis and are effective donors. Among a total of 225 studies, transgenic tobacco induces the accumulation of anthocyanin, delphinidin, and proanthocyanidin and the expression of *CHI*, *F3H*, *LAR*, and *ANR*. *Solanum* plants also show a significant increase in *F3′5′H*, *DFR*, *ANS*, and *UFGT* expression. This observation is an interesting phenomenon, and we should consider the importance of plant taxonomic characteristics on experimental design.

For most plants, regeneration and transformation remain arduously challenging even after more than 30 years of continuous work, and only a handful of species can be transformed (Altpeter et al., 2016; Maher et al., 2020). *Nicotiana* and *Arabidopsis* are easy to induce transformants, and to evaluate the function of *MYB* genes in anthocyanin biosynthesis (Shen et al., 2019; Zhou et al., 2020; Figueroa et al., 2021). The meta-analysis indicated that more than half of the studies ectopically overexpressed *MYB*, which significantly increased the contents of anthocyanin, delphinidin, proanthocyanidin and kaempferol and promoted the expression of *PAL*, *F3′5′H*, *ANS*, and *LAR*. In contrast, homologous expression in plants considerably enhanced the accumulation of cyanidin, flavonol, quercetin and DPPH and promoted the transcription of *C4H*, *4CL*, *FLS*, *F3′H*, *UFGT*, and *ANR*. Although, the expression systems of plants significantly enhanced the expression of *CHS*, *CHI* and *F3H*, heterologous or homologous expression demonstrated little difference in gene expressions. In conclusion, the reliability of homologous and heterologous overexpression should be considered in *MYB* transformation. The individual overexpression of *RrMYB5* and *RrMYB10* in *Rosa rugosa* transgenic shoots enhanced the accumulation of PAs and anthocyanins. Interestingly, in *RrMYB10*-transgenic tobacco, the contents of total anthocyanin and PA increased, while in *RrMYB5*-transgenic tobacco, the total anthocyanin content decreased and the PA content statistically increased (Shen et al., 2019). There may be differences in anthocyanin biosynthesis between heterologous expression and homologous expression. This result is interesting, but similar observations have been reported for WRKY and CBF/DREB TFs (Dong et al., 2017; Guo et al., 2019; Figueroa et al., 2021). These results also signify the importance of the expression systems of plants in experimental designs and results.

With the rapid advancement of plant genetic technology, transient transfection systems have been widely utilized for gene function analysis (Fan et al., 2020). Compared with a stable expression system, transient transformation provides an efficient, convenient and faster tool to analyze gene function (Wroblewski et al., 2005; Liao et al., 2019). To date, transient expression of *MYBs* has been well characterized in anthocyanidin biosynthesis of various plants, including rose (*Rosa hybrida*), radish (*Raphanus sativus*), *freesia* (*Freesia hybrida*), *gerbera* (*Gerbera hybrida*), apple (*Malus domestica*), tobacco (*Nicotiana tabacum*) and others (Shen et al., 2019; Fan et al., 2020; Li T. et al., 2020; Li Y. et al., 2020, Li et al., 2021; Zhong et al., 2020). In our studies, transient expression assays were undertaken in a dataset (105 studies) to test the function of MYB TFs. Delphinidin and kaempferol contents and the expression of *CHS*, *CHI*, *F3H*, *FLS*, *F3′H*, *F3′5′H*, *DFR*, *ANS*, and *UFGT* were significantly increased in the transient expression system, whereas anthocyanin, cyanidin, flavonol, proanthocyanidin and quercetin contents and *PAL* and *ANR* expression significantly increased in the stable expression system.

Therefore, transient gene transformation has a significant impact on flavonoid biosynthetic genes, while stable transformation causes better flavonoid accumulation. Accordingly, the use of transient transformation as a pattern system for appraising the function of *MYB* genes is obviously sustained in anthocyanidin biosynthesis, while caution should be used when extrapolating the outcomes generated with the transient system to other plants, particularly flavonoids accumulation. In addition, transient expression is only carried out in some tissues of the plant, and it is impossible to verify the effect of the *MYB* genes on the entire plant. This should be considered in future research. The number of gene transfers is closely related to anthocyanin biosynthesis in plants. The co-transformation of multiple genes enhanced anthocyanin accumulation than one or two genes (Liu et al., 2017; Sakai et al., 2019; Zhou et al., 2020).

This meta-analysis found that the transformation-induced enhancement was larger for the contents of anthocyanin and the expression of flavonoid biosynthetic genes when more than two genes were co-transferred to plants compared to transformation with a single gene. As shown in a multitude of studies, the flavonoid biosynthetic pathway is mainly controlled by two types of regulatory genes: R2R3-MYB modulatory genes, which can activate early biosynthetic genes, and ternary MBW complexes composed of MYB, bHLH and WD-repeat proteins, which can initiate late biosynthetic genes (Xu et al., 2015; Zhou et al., 2020; Yan et al., 2021). Consequently, it was reasonable to conclude that transferring three types of genes into plants might precisely have an additive influence on anthocyanin biosynthesis. This should be an important direction for further investigation.

Anthocyanins, the main secondary metabolites of plants, are a major subclass of flavonoids that are related to tissue colors and stress responses (Xu et al., 2015; Xia et al., 2021). Anthocyanin in plants is induced by various stresses, including light, cold, drought, wounding, and methyl viologen (Naing et al., 2018; Shen et al., 2019; Zhou et al., 2020; Ma et al., 2021). It was illustrated that the stresses could cause an increased accumulation of anthocyanidins in MYB transformation by enhancing the expression of flavonoid structural genes. In our study, flavonoid contents, especially anthocyanin, flavonol, and proanthocyanidin, were significantly increased in transgenic plants after stress treatment. Meanwhile, stresses also promoted DPPH activity of the transgenic plants, and MDA contents were significantly lower than in the wild type. Taken together, the accumulation of flavonoids in *MYB*-transgenic plants leads to the elevation of stress tolerance by enhancing the scavenging of excess ROS. Moreover, the overexpression caused an increase in the activity of DPPH and a decrease in MDA content, and transgenic plants showed a higher antioxidant capacity to improve plant stress tolerance. However, there are few studies on the *MYB* overexpression mechanism of activating anthocyanin regulation in plant stress response. This is another area for future research.

Thirty years of researches on the influence of overexpressing the MYB family for anthocyanin biosynthesis have generated an increasing body of data. However, the meta-analysis indicated that there are still various unanswered problems concerning the *MYB* induction of anthocyanin biosynthetic mechanism. In addition to anthocyanin biosynthesis, MYB transcription factors also regulate other plant secondary metabolism pathways, such as lignin, benzenoid, terpenoid, and glucosinolate pathways. However, other secondary metabolism pathways were rarely detected in transgenic plants. Therefore, how *MYB* transformation influences these metabolic pathways and how they play critical roles in plant growth and development needs further investigation. Besides, for different target traits, such as flower color, fruit color, and stem color, *MYB* overexpression needs to be used flexibly according to the accumulation of anthocyanins in different tissues and organs of different plants to achieve tissue specificity. In addition, how does *MYB* overexpression enhance stress tolerance in plants by causing the accumulation of anthocyanins and what is its mechanism are questions for serious discussion. Finally, we also need to find answer on how do MYB regulators participate in the flower coloration evolution and domestication of plants by directly regulating color changes?

## Data Availability Statement

The original contributions presented in the study are included in the article/Supplementary Material, further inquiries can be directed to the corresponding author/s.

## Author Contributions

QZ, TZ, and WL planned and designed the research. WL, TZ, and YY performed experiments. WL, YY, PL, LQ, and LL analyzed the data. JW and TC conducted fieldwork. WL wrote the draft manuscript. TZ and QZ finalized the manuscript. All authors read and approved the final manuscript.

## Conflict of Interest

The authors declare that the research was conducted in the absence of any commercial or financial relationships that could be construed as a potential conflict of interest.

## Publisher’s Note

All claims expressed in this article are solely those of the authors and do not necessarily represent those of their affiliated organizations, or those of the publisher, the editors and the reviewers. Any product that may be evaluated in this article, or claim that may be made by its manufacturer, is not guaranteed or endorsed by the publisher.

## Footnotes

1
http://getdata-graph-digitizer.com

## Abbreviations

PAL: phenylalanine ammonia lyase
C4H: cinnamate 4-hydroxylase
4CL: 4-coumarate-CoA ligase
CHS: chalcone synthase
CHI: chalcone isomerase
F3H: flavanone 3-hydroxylase
FLS: flavonol synthase
F3′H: flavonoid 3′ -hydroxylase
F3′5′H: flavonoid 3′, 5′ -hydroxylase
DFR: dihydroflavonol 4-reductase
ANS: anthocyanidin synthase
UFGT: UDP-flavonoid glucosyl transferase
LAR: leucoanthocyanidin reductase
ANR: anthocyanidin reductase
TFs: transcription factors
CPA1: cation/proton antiporter 1
CBF/DREB: C-repeat/dehydration-responsive element binding proteins
NAC: NAM/ATAF/CUC
TC: transformed plants
NC: non-transformed plants
CMA: Comprehensive Meta-Analysis
CI: confidence interval
CAT: catalase
MDA: malondialdehyde
DDPH: 1,1-diphenyl-2-picryl-hydrazyl.
