# Supplementary material for: Meta-Analysis of the Effect of Overexpression of MYB Transcription Factors on the Regulatory Mechanisms of Anthocyanin Biosynthesis
Source: Front Plant Sci. 2021 Dec 15;12:781343. doi: 10.3389/fpls.2021.781343 (PMC8714666; doi:10.3389/fpls.2021.781343)
Supplement: Supplementary file 1 [file Data_Sheet_1.pdf]

Table S1 The details of data collection in meta-analysis

To search for the keyword combination	
**Web of science**	**PubMed**
#1 TS= (anthocyanin* OR leucoanthocyanidin*) #2 TS= (overexpression* OR ectopic* expression* OR transgenic OR heterologous) #3 TS= （MYB OR MYB transcription factor*) ((#3) AND #2) AND #1	Search: (MYB OR MYB transcription factor*) AND (overexpression* OR ectopic* expression* OR transgenic OR heterologous) AND (anthocyanin* OR leucoanthocyanidin*) Filters: from 1950/1/1 - 2020/12/31

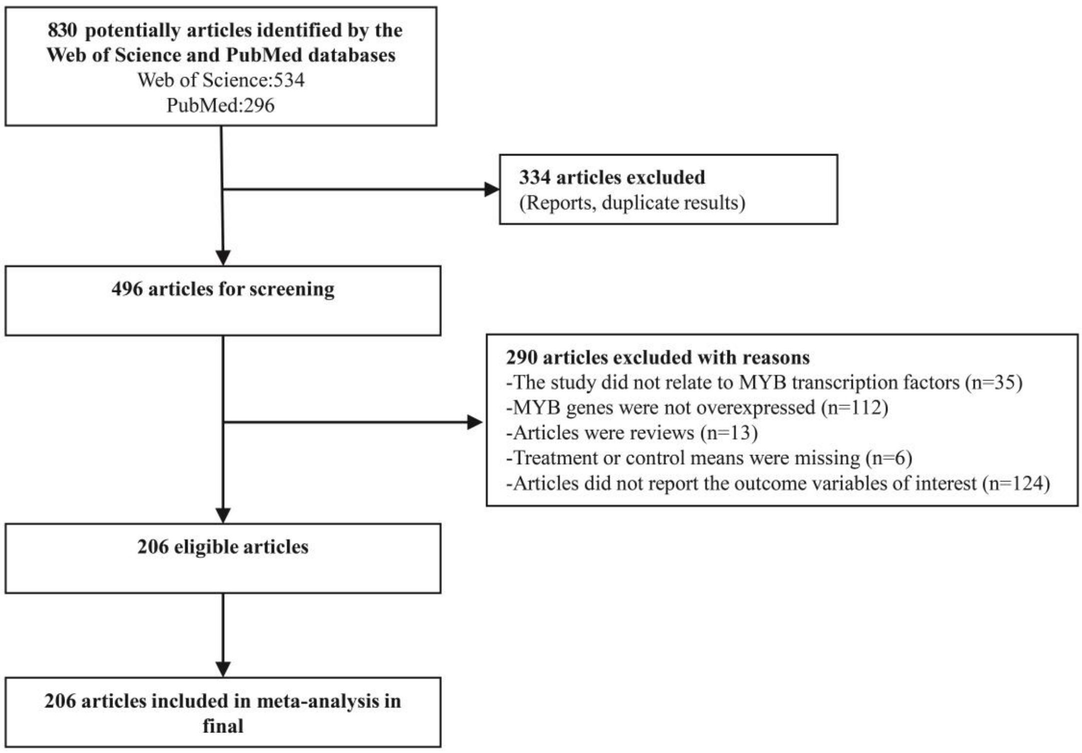

Fig. S1 Study selection flow chart

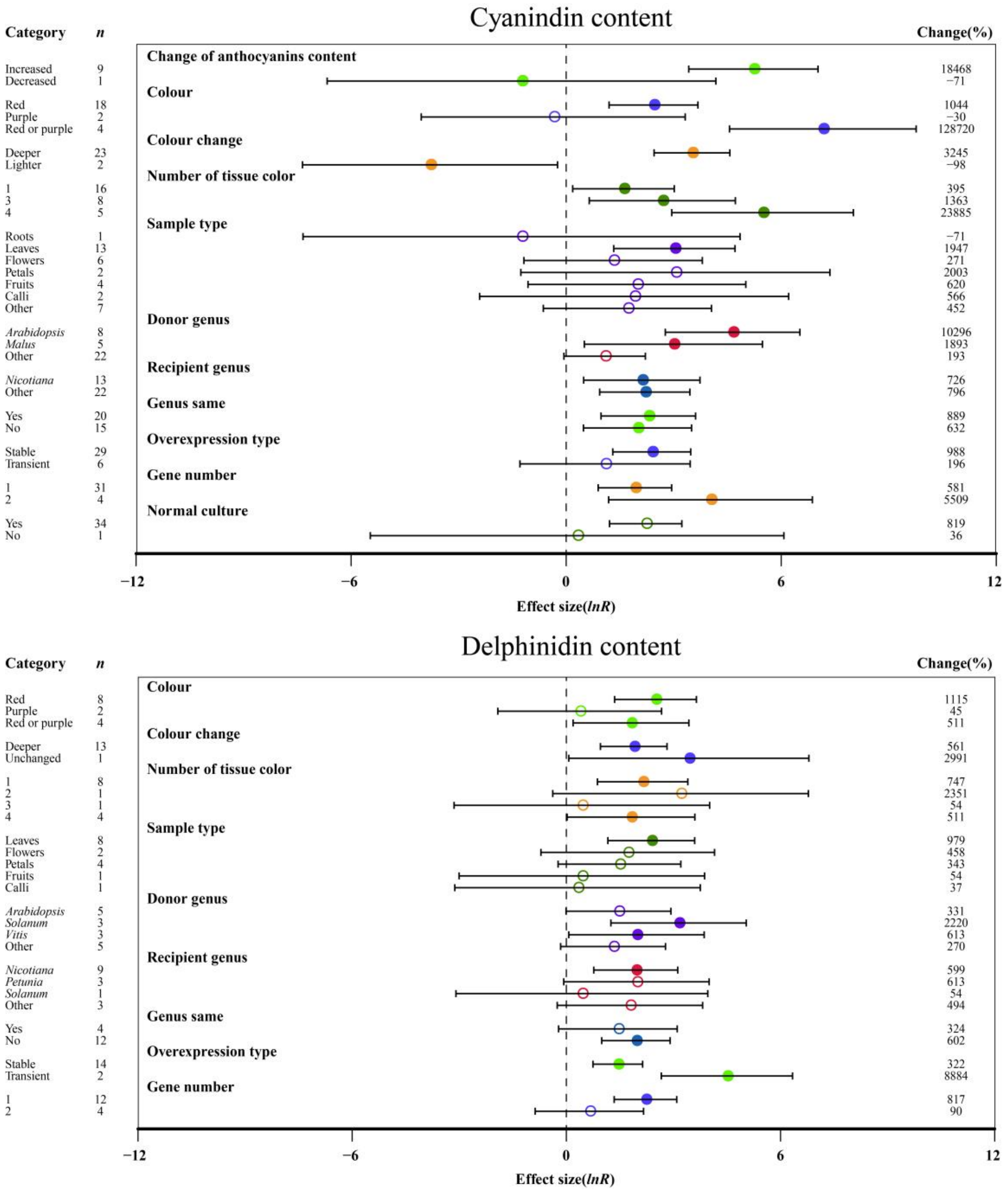

Fig. S2 Summary effects (ln *R*) of the influence of *MYB* overexpression on the content of cyanidin (S2-1) and delphinidin (S2-2) in plants. Ratios are unit-less. The ratios illustrate the strength of *MYB* overexpression on the summary effects of the content of cyanidin and delphinidin compared with the controls. Circles and horizontal bars indicate the summary effects and the 95% CI of eleven (S2-1; owing to insufficient studies, some moderators not analysed) and nine (S2-2; owing to insufficient studies, some moderators not analysed) moderator variables, respectively. *n* represents the number of studies. Subgroup list levels (subgroups or categories) of each moderator. The percent change (to the right of plots) refers to the raw percentage increase or decrease in the content of cyanidin and delphinidin induced by overexpression of *MYB*. The moderator variables of the examined plants are coloured and distinguished by different colours. Closed and open circles indicate the effects of significance and insignificance, respectively.

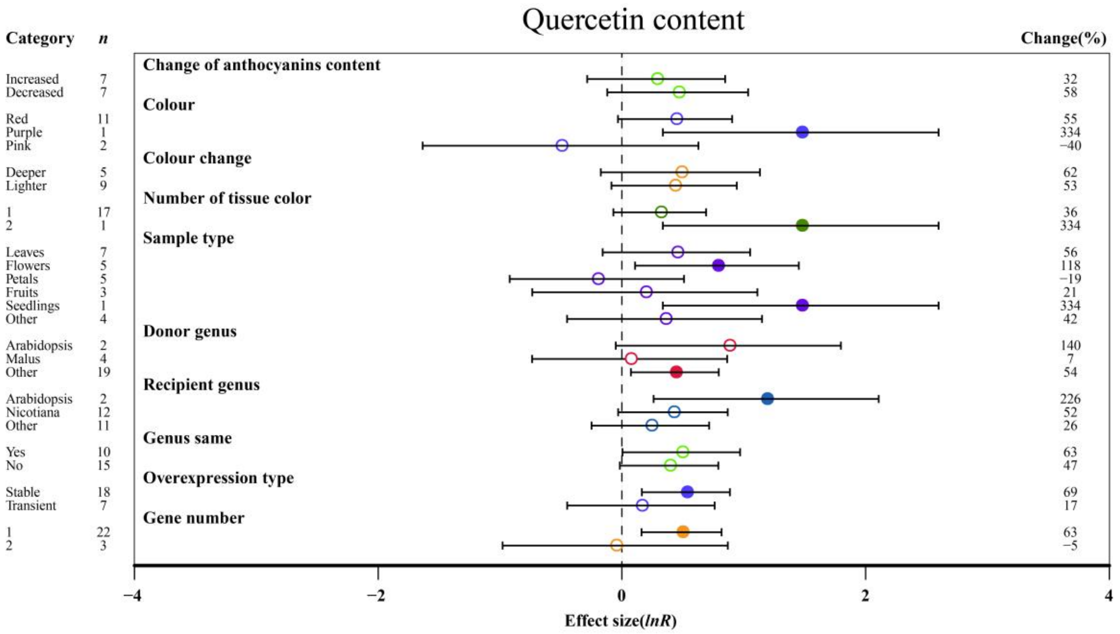

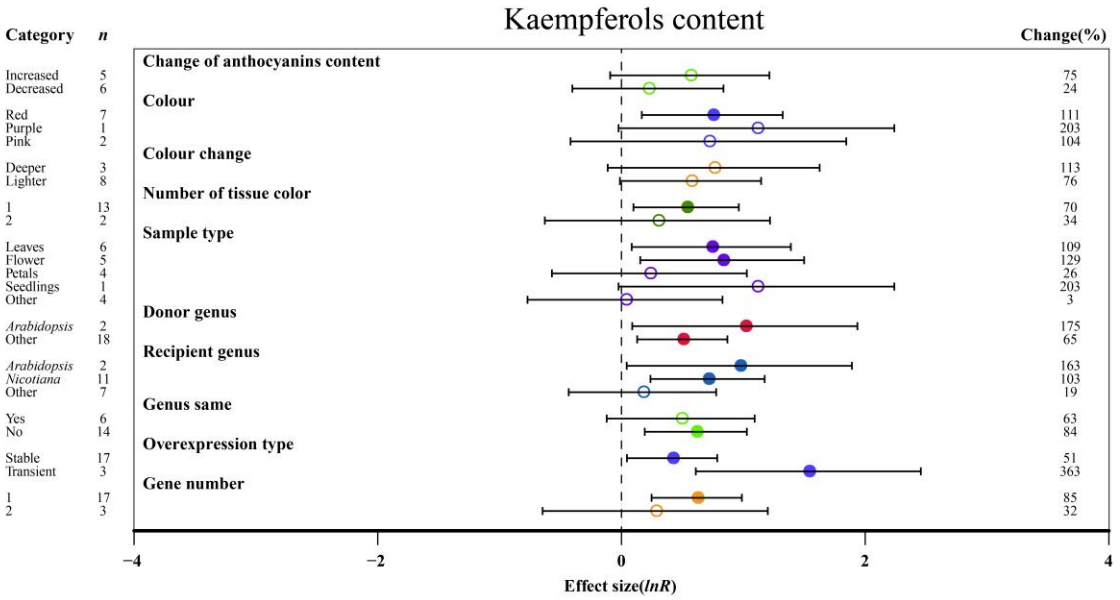

Fig. S3 Summary effects (ln *R*) of the influence of *MYB* overexpression on the content of quercetin (S3-1) and kaempferol (S3-2) in plants. Ratios are unit-less. The ratios illustrate the strength of *MYB* overexpression on the summary effects of the content of quercetin and kaempferol compared with the controls. Circles and horizontal bars indicate the summary effects and the 95% CI of ten (S3-1 and S3-2; owing to insufficient studies, some moderators not analysed) moderator variables, respectively. *n* represents the number of studies. Subgroup list levels (subgroups or categories) of each moderator. The percent change (to the right of plots) refers to the raw percentage increase or decrease in the content of quercetin and kaempferol induced by overexpression of *MYB*. The moderator variables of the examined plants are coloured and distinguished by different colours. Closed and open circles indicate the effects of significance and insignificance, respectively.

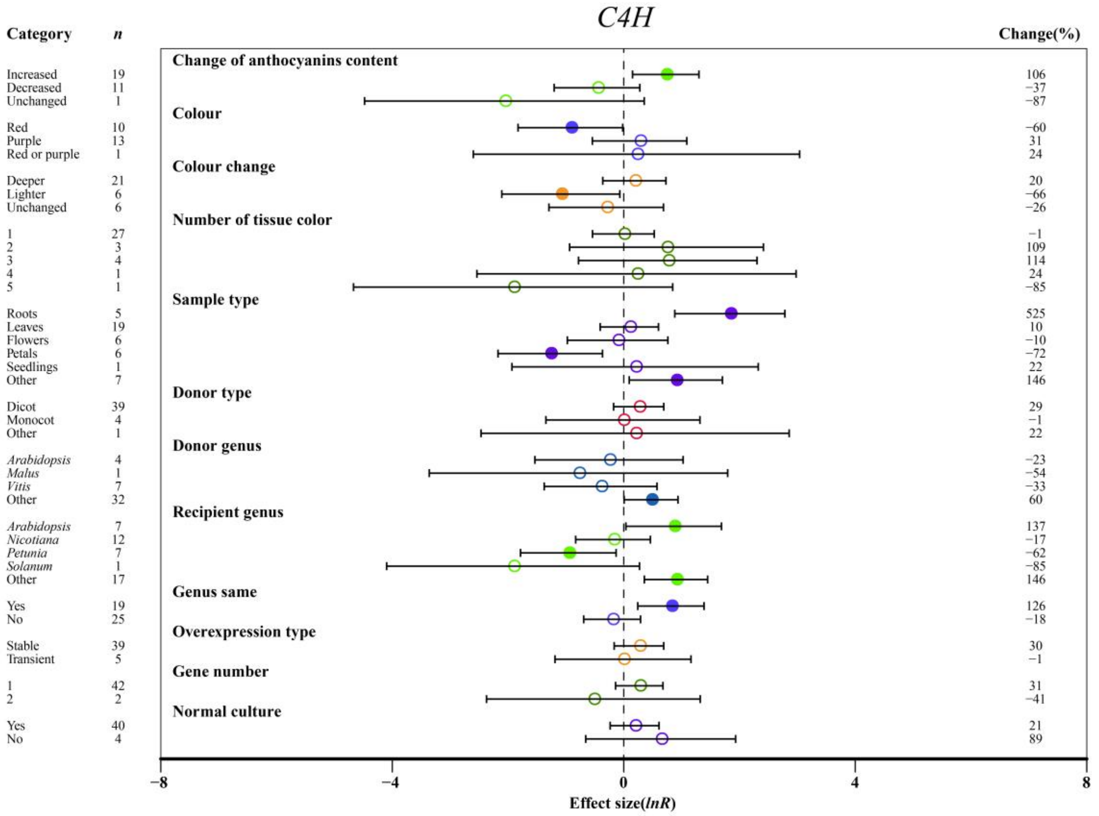

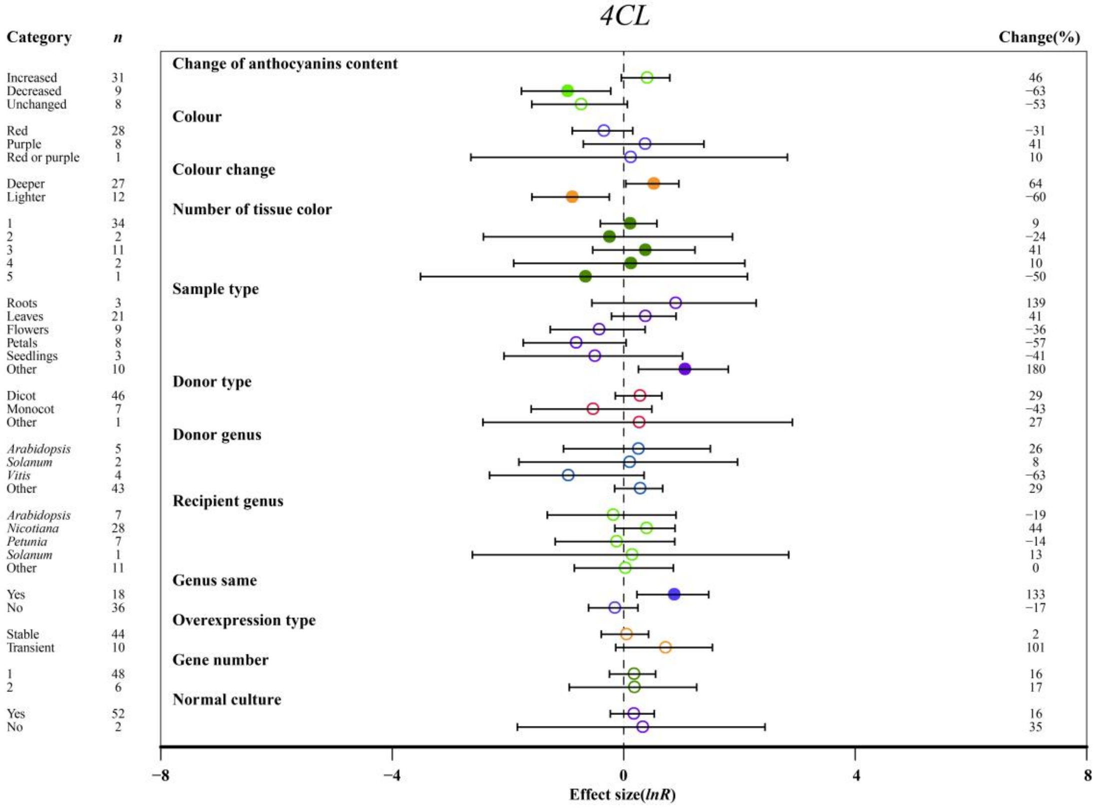

Fig. S4 Summary effects (ln *R*) of the influence of *MYB* overexpression on the expression of *C4H* (S4-1) and *4CL* (S4-2) in plants. Ratios are unit-less. The ratios illustrate the strength of *MYB* overexpression on the summary effects of the expression of *C4H* and *4CL* compared with the controls. Circles and horizontal bars indicate the summary effects and the 95% CI of twelve (S4-1 and S4-2; owing to insufficient studies, 'recipient type' not analysed) moderator variables, respectively. *n* represents the number

of studies. Subgroup list levels (subgroups or categories) of each moderator. The percent change (to the right of plots) refers to the raw percentage increase or decrease in the expression of *C4H* and *4CL* induced by overexpression of *MYB*. The moderator variables of the examined plants are coloured and distinguished by different colours. Closed and open circles indicate the effects of significance and insignificance, respectively.

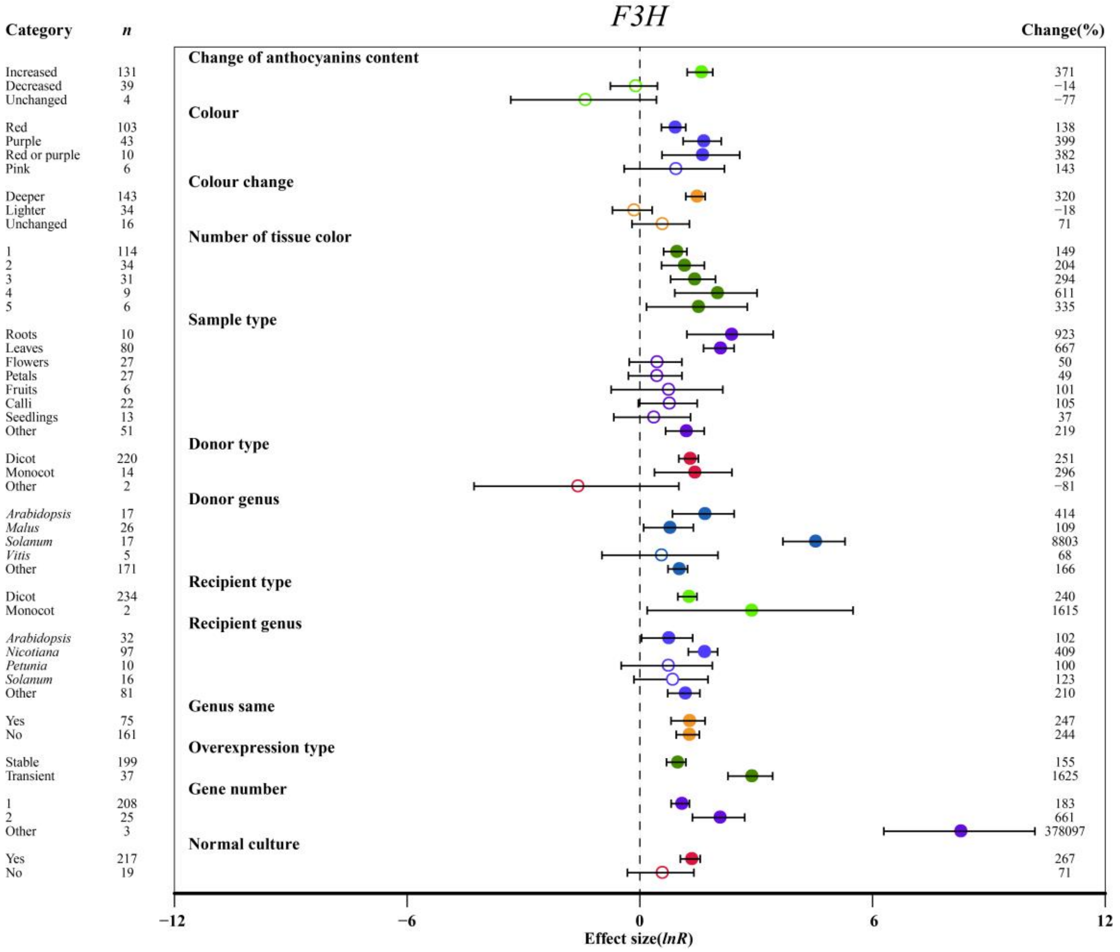

Fig. S5 Summary effects (ln *R*) of the influence of *MYB* overexpression on *F3H* expression in plants. Ratios are unit-less. The ratios illustrate the strength of *MYB* overexpression on the summary effects of *F3H* expression compared with the controls. Circles and horizontal bars indicate the summary effects and the 95% CI of thirteen moderator variables, respectively. *n* represents the number of studies. Subgroup list levels (subgroups or categories) of each moderator. The percent change (to the right of plots) refers to the raw percentage increase or decrease in *F3H* expression induced by overexpression of *MYB*. The moderator variables of the examined plants are coloured and distinguished by different colours. Closed and open circles indicate the effects of significance and insignificance, respectively.

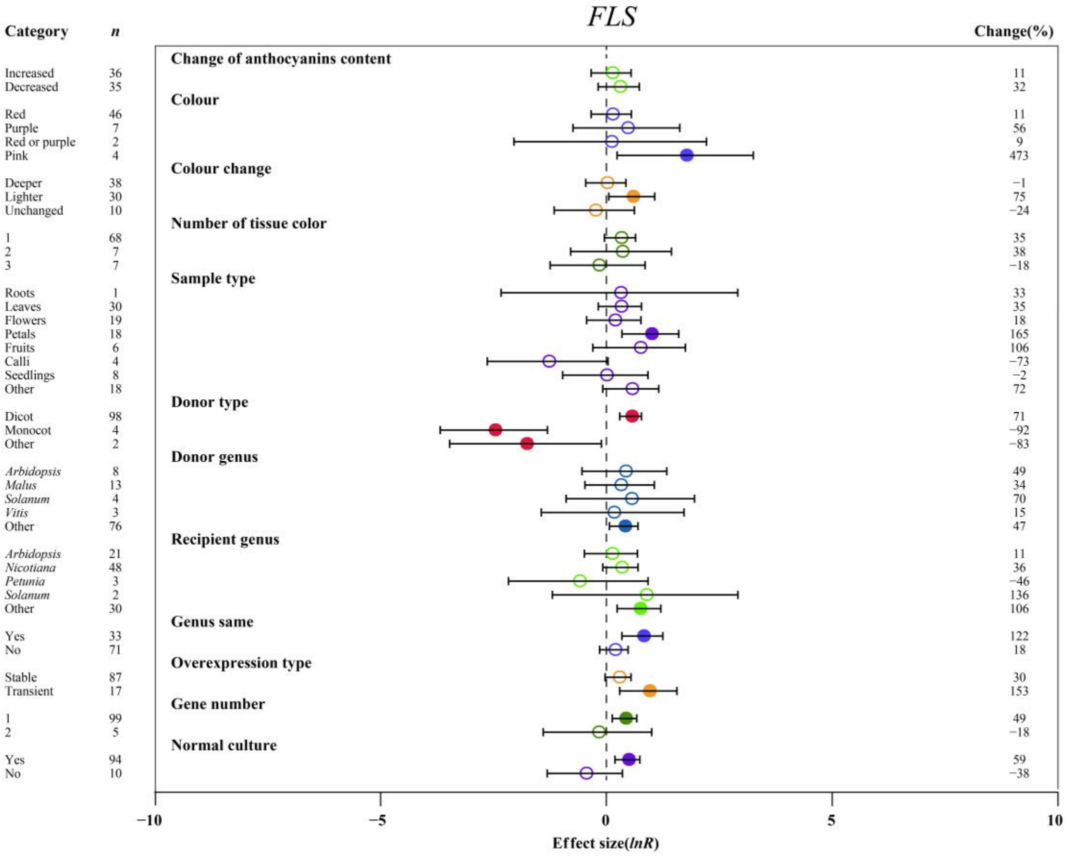

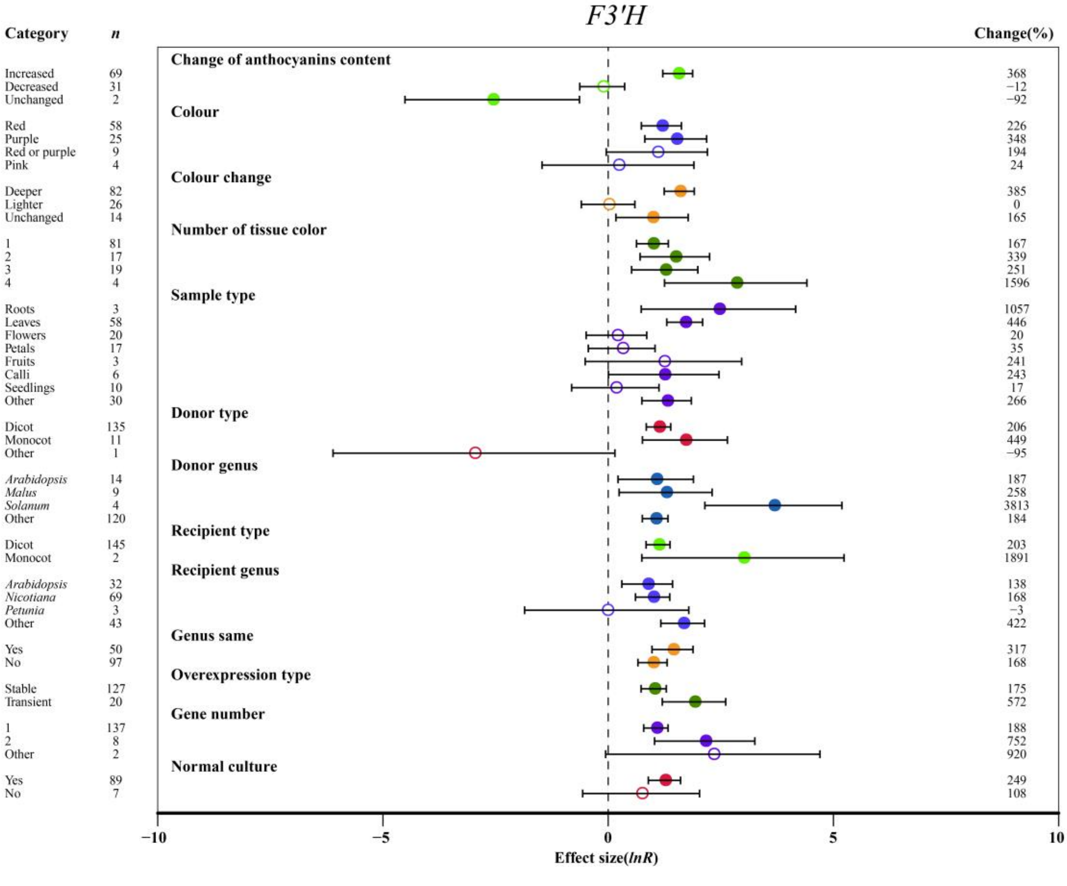

Fig. S6 Summary effects (ln *R*) of the influence of *MYB* overexpression on the expression of *FLS* (S6-1) and *F3'H* (S6-2) in

plants. Ratios are unit-less. The ratios illustrate the strength of *MYB* overexpression on the summary effects of the expression of *FLS* and *F3'H* compared with the controls. Circles and horizontal bars indicate the summary effects and the 95% CI of twelve (S6-1; owing to insufficient studies, 'recipient type' not analysed) and thirteen (S6-2) moderator variables, respectively. *n* represents the number of studies. Subgroup list levels (subgroups or categories) of each moderator. The percent change (to the right of plots) refers to the raw percentage increase or decrease in the expression of *FLS* and *F3'H* induced by overexpression of *MYB*. The moderator variables of the examined plants are coloured and distinguished by different colours. Closed and open circles indicate the effects of significance and insignificance, respectively.

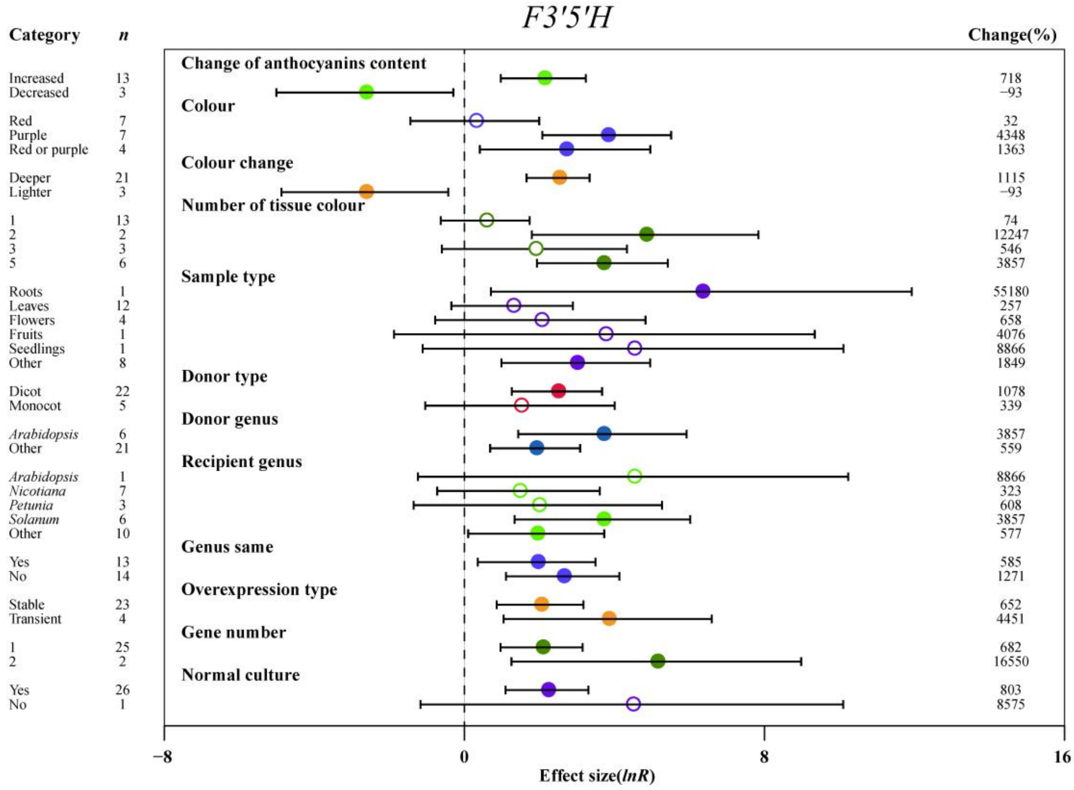

Fig. S7 Summary effects (ln *R*) of the influence of *MYB* overexpression on *F3'5'H* expression in plants. Ratios are unit-less. The ratios illustrate the strength of *MYB* overexpression on the summary effects of *F3'5'H* expression compared with the controls. Circles and horizontal bars indicate the summary effects and the 95% CI of eleven (owing to insufficient studies, some moderators not analysed) moderator variables, respectively. *n* represents the number of studies. Subgroup list levels (subgroups or categories) of each moderator. The percent change (to the right of plots) refers to the raw percentage increase or decrease in *F3'5'H* expression induced by overexpression of *MYB*. The moderator variables of the examined plants are coloured and distinguished by different colours. Closed and open circles indicate the effects of significance and insignificance, respectively.

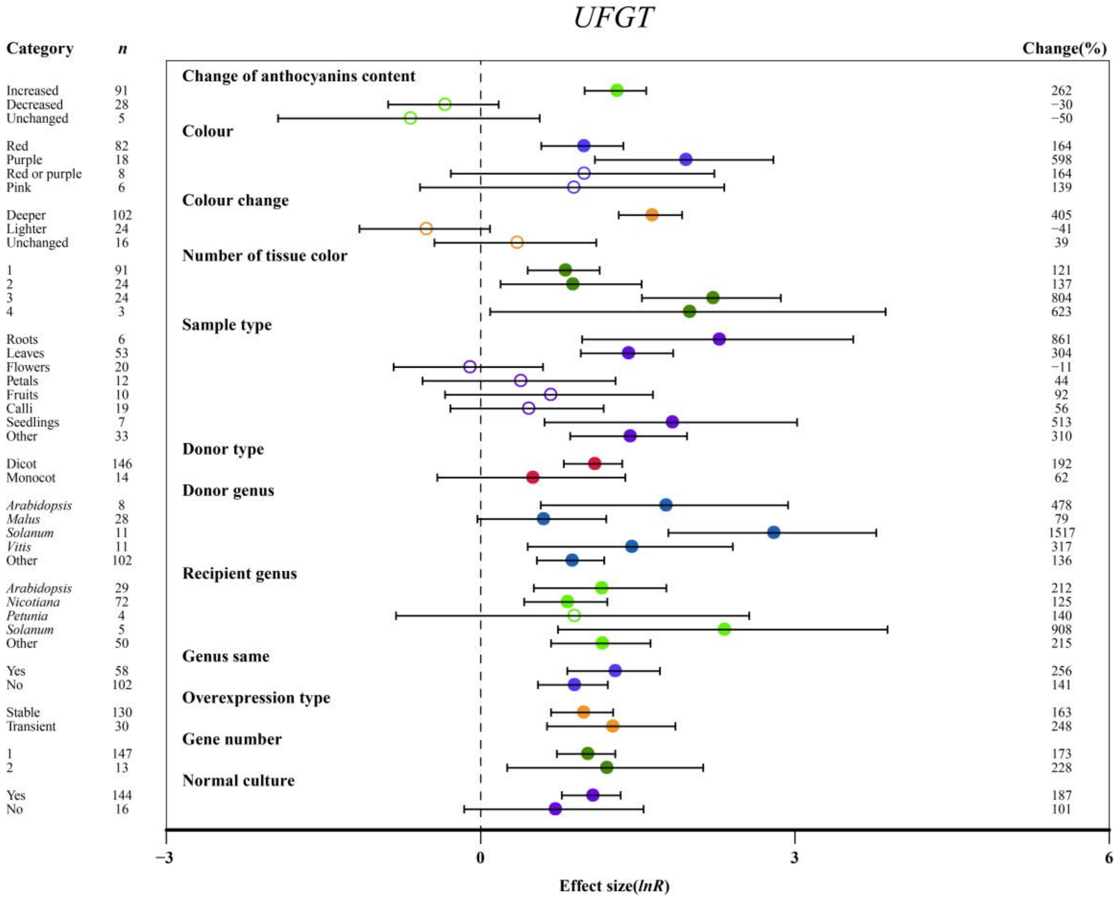

Fig. S8 Summary effects (ln *R*) of the influence of *MYB* overexpression on *UFGT* expression in plants. Ratios are unit-less. The ratios illustrate the strength of *MYB* overexpression on the summary effects of *UFGT* expression compared with the controls. Circles and horizontal bars indicate the summary effects and the 95% CI of twelve (owing to insufficient studies, 'recipient type' not analysed) moderator variables, respectively. *n* represents the number of studies. Subgroup list levels (subgroups or categories) of each moderator. The percent change (to the right of plots) refers to the raw percentage increase or decrease in *UFGT* expression induced by overexpression of *MYB*. The moderator variables of the examined plants are coloured and distinguished by different colours. Closed and open circles indicate the effects of significance and insignificance, respectively.
